# Ultra-processed food consumption and risk of chronic respiratory diseases mortality among adults: evidence from a prospective cohort study

**DOI:** 10.1007/s00394-024-03356-4

**Published:** 2024-02-28

**Authors:** Tefera Chane Mekonnen, Yohannes Adama Melaku, Zumin Shi, Tiffany K. Gill

**Affiliations:** 1 Adelaide Medical School, Faculty of Health and Medical Sciences, The University of Adelaide, South Australian Health and Medical Research Institute, Level 7, SAHMRI North Terrace, Adelaide, SA 5000 Australia; 2 Flinders Health and Medical Institute, Flinders University, Adelaide, South 5001 Australia; 3 Cancer Epidemiology Division, Cancer Council Victoria, Melbourne, VIC Australia; 4 Human Nutrition Department, QU Health, Qatar University, Doha, Qatar; 5 School of Public Health, College of Medicine and Health Science, Wollo University, 1145 Dessie, Ethiopia

**Keywords:** Ultra-processed food, Overall CRDs, COPD, Lung cancer, Mortality, Adults

## Abstract

**Purpose:**

The purpose of the study was to determine the relationships between ultra-processed food (UPF) consumption and risk of mortality due to chronic respiratory diseases (CRDs) overall, chronic obstructive pulmonary disease (COPD), and lung cancer.

**Methods:**

A total of 96,607 participants aged 55 years and over were included from the Prostate, Lung, Colorectal and Ovarian (PLCO) cancer trial. Dietary intake was measured using food frequency questionnaire. Cox regression was fitted to estimate the risk of all-cause mortality and mortality due to CRDs overall, COPD and lung cancer associated with UPF intake. Competing risk regression was used to account for deaths from other causes and censoring.

**Results:**

During the follow-up of 1,379,655.5 person-years (median 16.8 years), 28,700 all-cause, 4092 CRDs, 2015 lung cancer and 1,536 COPD mortality occurred. A higher intake of UPF increased the risk of mortality from CRDs overall by 10% (HR 1.10; 95% CI 1.01, 1.22) and COPD by 26% (HR 1.26; 95% CI 1.06, 1.49) but not associated with lung cancer mortality risk (HR 0.97; 95% CI 0.84, 1.12). However, the risk of lung cancer increased by 16% (HR 1.16; 95% CI 1.01, 1.34) in the highest UPF intake after multiple imputation. Dose–response relationships existed for CRDs and COPD mortality but not lung cancer.

**Conclusion:**

UPF consumption was associated with an increased risk of CRD mortality. The association between UPF consumption and lung cancer mortality is inconclusive and only significant when multiple imputation was applied.

**Supplementary Information:**

The online version contains supplementary material available at 10.1007/s00394-024-03356-4.

## Introduction

Non-communicable diseases (NCDs) are the leading cause of death worldwide, accounting for two-third of all deaths each year [1, 2]. The leading specific causes of deaths are cardiovascular diseases (CVDs), respiratory disease, cancer, and diabetes [2]. Chronic respiratory diseases (CRDs) are diseases of the respiratory tract including the lung [3] and are among the most prominent causes of disability and mortality globally [4].

Unhealthy diet together with tobacco use, physical inactivity and harmful use of alcohol, has been attributed to the growing burden of NCDs including CRDs [2]. A western dietary pattern, mostly consists of ultra-processed food (UPF), is associated with morbidity and mortality of NCDs [5, 6]. Based on the NOVA classification system, a commonly used tool that considers the nature (physical, biological, and chemical techniques used), extent and reasons for food processing, UPF are palatable, cost-effective, readily accessible, and energy dense [7]. They are also typified by their high content of saturated and hydrogenated fats, a dearth of dietary fiber, and substandard nutritional quality. Manufactured extensively via several industrial methods, such as hydrogenation, extrusion, and pre-frying [7], these foods may incorporate food additives and deleterious neoformed contaminants. These contaminants/additives can include acrylamide, titanium dioxide, bisphenols, phthalates, heterocyclic amines, polycyclic aromatic hydrocarbons, furans, and emulsifiers. Intriguingly, these elements have been associated with various health risks including inflammation, carcinogenesis, genotoxic effects, and disruptions in the gut microbiota [8–12].

The association of UPF consumption with CVD, digestive tract disorders, cancer, depression, and all-cause mortality have been well documented [7, 12–17]. However, evidence on the risk of mortality from the underlying causes of CRDs and consumption of UPF is very limited. A recent study using UK Biobank data found a positive association of UPF consumption and respiratory disease but not mortality. However, the study was underpowered due to relatively lower numbers of respiratory mortality (793 CRDs deaths) [18]. Other cross-sectional studies from Brazil have reported that a higher consumption of UPF was associated with increased risk of asthma and wheezing in paediatric and adolescent population [19, 20]. As a result of this limited evidence on the relationship between UPF consumption and CRDs, further studies have been recommended which focus on greater characterization of UPF, relationships and the potential mechanisms linked with risk of CRDs [17]. The current study aimed at exploring the association between UPF intake and mortality caused by overall CRDs, chronic obstructive pulmonary diseases (COPD) and lung cancer using the Prostate, Lung, Colorectal and Ovarian cancer trial (PLCO) data.

## Materials and methods

The current study is reported in accordance with the Strengthening the Reporting of Observational Studies in Epidemiology-Nutritional Epidemiology (STROBE-nut), an extension of STROBE statement [21] and conforms to the International Committee of Medical Journal Editor’s recommendation.

### Study population

This study used data from the PLCO, a multi-center, two arm, randomized trial comprising ten study centers (Birmingham, Denver, Detroit, Honolulu, Marshfield, Minneapolis, Pittsburgh, Salt Lake City, St Louis, and Washington) in the USA. The PLCO trial study objective and design are described in detail elsewhere [22] but briefly, the aim of the PLCO trial was to determine the effects of cancer screening on cancer-related mortality and secondary endpoints (disease-free survival, duration of response and incidence) in adults. For example, annual screening with chest radiography was carried out for participants in the intervention group to examine the effect of screening on lung cancer mortality compared to participants who received the usual service. Between November 1993 and September 2001, 154,887 adults aged 55–74 years were included in the trial. The participants were selected based on the prespecified inclusion and exclusion criteria. Equal proportions of individuals were allocated to the intervention arm and control arm (those who followed the standard health care services) [22]. The PLCO trial was ethically approved by the US National Cancer Institute Review Boards and written informed consent was obtained from each participant [22].

In the current study, those who (1) didn’t return the baseline questionnaire [n = 4918]; (2) had not completed the dietary history questionnaire (DHQ) [n = 33,241]; (3) had an invalid DHQ (including those who did not have a date of DHQ completion, died before completing DHQ, had eight or more missing/multiple frequency responses on DHQ and implausible energy intake (the bottom and top 1% of dietary energy intake)) [n = 10,437] and (4) past history of any cancer [n = 9,684] were excluded. A total of 96,607 participants were included in the analysis (Supplementary Fig. 1). To assess non-participation bias due to the high number of participant exclusions, we compared the standardized mean differences of baseline characteristics of included participants with those excluded. There were no statistically significant variations observed between the two groups.

### Dietary assessment

Dietary intake was assessed using a self-administered food frequency questionnaire (FFQ), the DHQ version 1.0, which was developed by staff at the Risk Factor, Monitoring and Methods Branch, National Cancer Institute [23]. The FFQ, which comprising 156 questions, was introduced in 1998 to both recruitment arms within a median timeframe of three years following the random allocation of study participants. It consisted of a one-year recall on foods and beverages intake relating to frequency, serving size, type, seasonal intakes, cooking methods, fat uses, low-fat diet use, summary questions and nutrient supplementations [23, 24]. The DietCalc software was used to estimate energy and nutrient intake by linking with the national nutrition databases for the USDA’s 1994–96 Continuing Survey of Food Intakes by Individuals (CSFII) and the Nutrition Data Systems for Research (NDSR) [25, 26]. The DHQ has been validated and provides better nutrient estimates compared to the 1992 Block FFQ, the 1995 Block FFQ and the Willett FFQ [24]. The Eating at America’s Table Study (EATS) validated the DHQ against four 24-h dietary recalls among 1644 nationally representative participants and found that the correlation coefficients of nutrients intake ranged from 0.51 to 0.78 in women and 0.41 to 0.83 in men [24]. A self-administered FFQ has also been showed to have moderate validity for various food groups such as fruits, egg, meat, nuts, alcoholic and non-alcoholic beverages, salty snacks, tea, and coffee [27]. Therefore, the FFQ is an appropriate tool to assess and characterize usual dietary intakes (both nutrients and food groups) of adults in epidemiological studies.

All foods and beverages consumed by study participants were categorised into four food groups by two experts (TCM and YAM) based on the NOVA food classification definitions which consider the purpose, nature, and the extent of food processing. The agreement in classification of foods by the two individuals were checked by a senior nutritional epidemiologist (ZS). All foods were classified into one of the four groups: (1) unprocessed or minimally processed, (2) processed culinary ingredients, (3) processed foods and (4) ultra-processed foods (UPF). The current study defined UPF as described by previously published studies on the association between UPF consumption and health outcomes [28, 29]. The total number of foods and beverages, 275 in total, had their gram amounts determined based on each question about food frequency and serving size. Out of these, 145 were categorized as UPF. When calculating the DHQ gram variables, it was found that more than one question contributed to the gram amount for a particular food. Generally, food items were categorised as UPF if they were carbonated drinks, savory packaged snacks; ice cream, chocolate, confectionery; mass-produced packaged breads and buns; margarines and spreads; industrial cookies, pastries, cakes, and cake mixes; breakfast ‘cereals’, ‘cereal’ and ‘energy’ bars; ‘energy’ drinks; flavoured milk drinks; cocoa drinks; sweet desserts made from fruit with added sugars, artificial flavours and texturizing agents; cooked seasoned vegetables with ready-made sauces; meat and chicken extracts and ‘instant’ sauces; powdered or ‘fortified’ meal substitutes; pre-prepared pies, pasta and pizza dishes; poultry and fish ‘nuggets’ and ‘sticks’, sausages, burgers, hot dogs, and reconstituted meat products, and instant soups, and noodles (Supplementary Table 1).

The proportion of UPF consumption in the diet (% weight/day) was estimated by dividing the sum of all UPF items (in gram/day) to the total daily dietary intake of individuals (the sum of all food items of the four NOVA groups). The percent of weight ratio was further categorized into quintiles. For comparison and face-validity with published findings, we also calculated the percentage contribution of UPF consumption in the total dietary energy (% kcal/day). The main result of this study was compiled using percent of UPF consumption in weight ratio because percent weight ratio accounts foods that do not provide energy such as artificially sweetened drinks and non-nutritional factors mixed during food processing (additives, neoformed contaminants and other by-products) [30, 31].

### Assessment of other covariates

Participants’ baseline demographic characteristics such as sex, age, ethnic background, marital status, education, occupational status, study arm and family history of lung cancer, lifestyle factors and medical history including cigarette smoking (never, current and former smoker), body mass index (BMI), history of hypertension, diabetes mellitus, heart attack, stroke, colon comorbidity, liver morbidity, emphysema, chronic bronchitis, aspirin and ibuprofen use were measured using baseline questionnaire (BQ). Physical activity (minutes/session) and family income were measured by supplementary questionnaire (SQ) in 2006/2007. BMI was determined by the ratio of weight (in kg) to height (in m^2^) and categorised into four groups as underweight (BMI < 18.5), normal (BMI ≥ 18.5 and ≤ 24.99), overweight (BMI ≥ 25 and ≤  29.99) and people with obesity (BMI ≥ 30). Average family income was grouped into three categories: < $50,000, between $50,000–$99,000 and ≥ $100,000. Physical activity was measured in terms of the total time spent (in minutes) during each session for moderate-to-strenuous exercise documented from the self-reported SQ. Age at completion of DHQ, total energy intake (kcal/day), Healthy Eating Index-2015 (HEI) and alcohol consumption, which is the sum of alcohol from beer, wine, and liquor, were assessed from DHQ. Date of completion and outcome occurrence (between 1998 and 2018) were found in the DHQ and brief survey questionnaire, respectively.

### Ascertainment of outcomes

The outcomes of the current study were risk of mortality from overall CRDs, COPD and lung cancer. Mortality status was ascertained through an annual review update form and mortality is also linked periodically to the US National Death Index. The PLCO trial used the International Classification of Diseases, ninth Revision (ICD-9) to define the underlying causes of mortality, from death certificates: lung cancer (code: 162- malignant neoplasms of trachea, bronchus, and lung), COPD and allied conditions (code: 490–496 includes bronchitis not specified as acute or chronic, chronic bronchitis, emphysema, asthma, bronchiectasis, extrinsic allergic alveolitis and chronic airways obstruction, not elsewhere classified). In this study overall CRD mortality referred to any death caused by CRDs including COPD, malignancy of trachea, bronchus, and lung, diseases of mediastinum and pleura, and all other unspecified as acute or chronic diseases of respiratory system. Mortality from acute respiratory diseases were excluded.

### Statistical analysis

After computing the contribution of the proportion of UPF consumption, participants’ baseline characteristics were compared by quintile of UPF consumption using Chi-Squared tests for categorical variables (proportion) and analysis of variance for continuous covariates (mean). We used Cox proportional hazard model to determine the association of proportion of UPF consumption and mortality due to overall CRDs, COPD, and lung cancer. The main exposure variable, UPF consumption was fitted to the Cox regression model as a categorical (quintiles) and as continuous variable (per 5% absolute increment in the proportion of UPF consumption). Study participants contributed to person-years until the date of completion of DHQ, diagnosis of lung cancer, death, or the last date of completion of the brief survey questionnaire.

Models were adjusted for a range of potential confounders defined a priori. As recommended, we identified these confounders based on the available literature, rather than relying solely on statistical criteria [32, 33]. Initially, we conducted an unadjusted model by including UPF intake and the risk of outcomes in the model without other covariates. Subsequently, we adjusted for age and sex to examine their impact on the association. Finally, in the multivariable-adjusted models, we assessed the association between UPF intake and CRDs for each outcome by adjusting for age (in years), sex (men vs women), study arm (intervention vs control), ethnicity (Hispanic vs non-Hispanic), marital status (married, widowed, divorced, separated/never married), education (up to high school, post-high school training, college graduate), occupation (homemaker, employed, retired), family history of lung cancer (yes, no), smoking (never, current, former), BMI (< 18.5, 18.5–24.99, 25–29.99, ≥ 30 kg/m^2^), dietary energy (kcal/day), alcohol consumption (g/day), diabetes (yes, no), hypertension (yes, no), chronic bronchitis (yes, no), emphysema (yes, no), heart attack(yes, no), and stroke (yes, no).

However, residual confounding can not be ruled out. Thus the e-value was determined to assess the minimum strength of association, on the risk ratio scale, which an unmeasured confounder would need to have with both the exposure and outcome to fully explain away the UPF-outcome association, conditioned by the measured covariates using the package ‘EValue’ version 4.1.3 in R-software [34].

We determined the linear trend across the quintiles of UPF consumption and in relation to each mortality cause and investigated the assumptions of proportionality of the Cox regression using a global test of Schoenfeld residuals for non-significant values (all *p-value* > *0.05*). A dose–response analysis for the non-linear relationships between the proportion of UPF consumption and the underlying cause of mortality from CRDs overall, COPD and lung cancer was determined using a restricted cubic spline with five knots (5th, 27.5th, 50th, 72.5th and 95th percentile). A 5% increment in UPF intake was taken as a benchmark because of the lowest significant association was observed at this value. We tested the p-value for non-linearity by making the coefficients of regression for the middle splines equal to zero [35].

To check the effect of heterogeneity between subgroups on the association of UPF consumption and mortality, subgroup analysis was done by stratifying the data using sex (men/women), age (55–59, 60–64, 65–69, ≥ 70), smoking (never, current, former), BMI (< 25, 25–29.99, ≥ 30) hypertension (yes/no), diabetes (yes/no), chronic bronchitis (yes/no), and emphysema (yes/no). Multiplicative interactions were checked using the p-value for interaction, which resulted from the likelihood ratio test [36].

The cause-specific hazard estimates obtained from Cox proportional regression models may be impacted by competing risks. To overcome this, we used the cumulative incidence function and Fine-Gray’s competing risk regression to estimate the marginal probability of competing events and sub-distributional hazards respectively. In contrast to Kaplan Meier and Cox models, the Fine-Gray competing risk regression provides a better estimation for the risk of the main outcome of interest when one or more competing risks are presented [37].

### Sensitivity analysis

The following sensitivity analyses were performed after generating the final model: (1) including covariates with missing values such as family income and physical activity (which had 28% and 24% of values that were missing, respectively) and other covariates with missing values (Supplementary Table 2) in the regression model using multiple imputation (MI). MI was performed using multivariate imputation by chained equation, the MICE Methods [38] (20 data sets imputed) for covariates with missing values. MI is preferred and more efficient than complete case when data are missing at random but when data are missing completely at random, both MI and complete-case analysis have negligible bias. In general, complete case analysis is biased towards the null when data are missing at random and has negligible bias when missingness is independent of the outcome. However, when missingness is independent of the outcome, MI is biased away from the null [39]. Given these and smaller standard error (Se) in the final models for complete-case analyses than MI (e.g. Se: 0.072 vs 0.083 for highest UPF category of lung cancer mortality), we presented the main findings using complete-case analyses. (2) participants with baseline diabetes, hypertension, stroke, emphysema, obesity, and heart attack were excluded to explore the potential effects on cause-specific hazard estimates; (3) excluding deaths that occurred during the first 5 years of follow-up for lung cancer, COPD and overall CRDs mortality to evaluate if the observed association resulted from reverse causation; (4) excluding deaths from lung cancer and COPD from all-cause mortality and overall CRDs mortality; (5) additionally adjusted for nutritional factors that are highly linked to health outcomes [30]. These variables included: alcohol consumption, dietary sodium, total fat, trans-fatty acid, fiber, and polyunsaturated fatty acid (PUFA) intake. In the analysis, nutrient intake was adjusted for energy using residual method. (6) We also examined the association between overall UPF consumption (gm/day) and risk of mortality from CRDs overall, COPD and lung cancer by adjusting for each subgroup of UPF, fish, fruits, and vegetable intake. The individual contributing foods of UPF were further categorized into seven subgroups (animal-based processed foods; artificial and sugar-sweetened beverages; salads, spreads, and sauces; milk shakes, sweets, and condiments; quick breads, ready-to-eat/heat grains; cookies, and savoury foods; and other UPF intake) (Supplementary Table 1). Besides for each subgroup of UPF intake, fish, fruits and vegetable intake, models were also adjusted for age, sex, study arm, ethnicity, marital status, education, occupation, smoking status, BMI, family history of lung cancer, history of comorbidities, alcohol intake. Additionally, we further examine the association between UPF intake and risk of mortality due to CRDs overall, COPD and lung cancer by accounting the intensity of current and former cigarette smokers (never, currently smokes 1–10 cigarettes/day, currently smokes 11–20 cigarettes/day, currently smokes 21+ cigarettes/day, former smoker who quit less than 10 years ago, 11–20 years ago, more than 20 years ago) [40].

A secondary analysis on the association of contribution of UPF in energy ratio and mortality from overall CRDs, COPD and lung cancer was undertaken to compare the robustness of the findings with the current and already available studies. A hazard ratio (HR) with 95% of confidence interval (CI) and a *p-value* < *0.05* were used to declare statistical significance and all tests are two-sided. All analyses were done using R statistical software (version 4.2.2).

## Results

### Characteristics of participants

Among the total participants, 53% (50,803/96,607) were female and the mean (SD) age was 65.6 (5.7) years (Table 1). The mean (SD) proportion of UPF consumption in the total diet (gm/day) was 31.2% (14%) and ranged from 13.4% (4%) to 52.7% (8.2%) across quintiles. The mean (SD) energy contribution of UPF consumption was found to be 37.1% (11.2%) of the total dietary energy (% kcal/day). Participants in the fifth quintile (highest consumption) were younger compared to the first quintile of UPF consumption. A higher proportion of people who had BMI ≥ 30 (27.8% vs 18.1%), diabetes (7.8% vs 6.2%), emphysema (2.4% vs 1.7%), hypertension (34.6% vs 30.1%) and were current smokers (12.8% vs 6.3%) was found in the highest quintile compared to the lowest quintile of UPF consumption (Table 1).

**Table 1:** Distribution of baseline characteristics by quintiles of proportion of UPF consumption in older adults enrolled in the PLCO trial in the USA

	Overall	Proportion of UPF (% weight in the diet)					
		Q1 0.01 to 18.8	Q2 18.81 to 26.3	Q3 26.31 to 33.8	Q4 33.81 to 42.7	Q5 42.71 to 99	p-for trend^a^
Demographic and lifestyle variables							
No	96,607	19,622	19,440	19,482	19,552	18,511	
Sex = Females, n (%)	50,803 (52.6)	13,044 (66.5)	10,872 (55.9)	9838 (50.5)	8991 (46.0)	8058 (43.5)	< 0.001
Trial arm = Controls, n (%)	47,428 (49.1)	9563 (48.7)	9545 (49.1)	9551 (49.0)	9507 (48.6)	9262 (50.0)	0.053
Ethnicity, n (%)							< 0.001
Non-Hispanic White	88,013 (91.1)	17,352 (88.4)	17,708 (91.1)	18,029 (92.5)	18,105 (92.6)	16,819 (90.9)	
Non-Hispanic Black	3061 (3.2)	485 (2.5)	552 (2.8)	554 (2.8)	597 (3.1)	873 (4.7)	
Hispanic	1374 (1.4)	321 (1.6)	290 (1.5)	250 (1.3)	255 (1.3)	258 (1.4)	
Asian, Pacific Islander and American Indian	4124 (4.3)	1453 (7.4)	886 (4.6)	647 (3.3)	585 (3.0)	553 (3.0)	
Age (in years), Mean (SD)	65.6 (5.7)	66.0 (5.7)	65.9 (5.7)	65.8 (5.7)	65.6 (5.7)	64.6 (5.7)	< 0.001
Education, n (%)							< 0.001
Up to high school or less	27,987 (29.0)	4985 (25.5)	5352 (27.6)	5696 (29.3)	5956 (30.5)	5998 (32.4)	
Post-high school training	33,139 (34.4)	6605 (33.7)	6497 (33.5)	6655 (34.2)	6722 (34.5)	6660 (36.0)	
College graduate	35,296 (36.6)	7996 (40.8)	7559 (38.9)	7089 (36.5)	6823 (35.0)	5829 (31.5)	
Marital status, n (%)							< 0.001
Married	75,771 (78.6)	14,781 (75.5)	15,345 (79.1)	15,644 (80.5)	15,699 (80.5)	14,302 (77.4)	
Widowed	7837 (8.1)	1915 (9.8)	1592 (8.2)	1464 (7.5)	1446 (7.4)	1420 (7.7)	
Divorced	9095 (9.4)	2019 (10.3)	1737 (8.9)	1646 (8.5)	1700 (8.7)	1993 (10.8)	
Separated/never married	3732 (3.9)	872 (4.5)	734 (3.8)	690 (3.5)	666 (3.4)	770 (4.2)	
Occupation, n (%)							< 0.001
Homemaker	11,592 (12.1)	2900 (14.9)	2590 (13.4)	2344 (12.1)	2140 (11.0)	1618 (8.8)	
Employed	38,340 (39.9)	7325 (37.5)	7349 (38.0)	7398 (38.2)	7926 (40.7)	8342 (45.2)	
Retired	41,624 (43.3)	8437 (43.2)	8582 (44.3)	8733 (45.0)	8457 (43.5)	7415 (40.2)	
Others	4617 (4.8)	862 (4.4)	837 (4.3)	916 (4.7)	940 (4.8)	1062 (5.8)	
BMI at baseline, n (%)							< 0.001
< 18.5	648 (0.7)	217 (1.1)	125 (0.7)	114 (0.6)	111 (0.6)	81 (0.4)	
18.5–24.99	32,585 (34.2)	8285 (42.8)	7025 (36.6)	6427 (33.4)	5897 (30.6)	4951 (27.1)	
25–29.99	40,538 (42.5)	7331 (37.9)	8077 (42.1)	8410 (43.7)	8564 (44.4)	8156 (44.6)	
≥ 30	21,592 (22.6)	3510 (18.1)	3979 (20.7)	4299 (22.3)	4722 (24.5)	5082 (27.8)	
Cigarette smoking, n (%)							< 0.001
Never smoker	46,560 (48.2)	9877 (50.3)	9599 (49.4)	9357 (48.0)	9437 (48.3)	8290 (44.8)	
Current smoker	8617 (8.9)	1239 (6.3)	1476 (7.6)	1647 (8.5)	1888 (9.7)	2367 (12.8)	
Former smoker	41,417 (42.9)	8502 (43.3)	8363 (43.0)	8476 (43.5)	8222 (42.1)	7854 (42.4)	
Family income (n = 69,978)							< 0.001
< $50,000	35,086 (50.2)	6888(47.8)	6894 (48.5)	7108 (50.1)	7262 (51.4)	6934 (53.2)	
$50,000–99000	19,530 (27.9)	3961 (27.5)	4052 (28.5)	4061 (28.6)	3923 (27.8)	3533 (27.1)	
≥ $100,000	6299 (9.1)	1422 (9.9)	1341 (9.4)	1270 (9.0)	1198 (8.5)	1068 (8.2)	
Refused to report	9063 (13.0)	2148 (14.9)	1933 (13.6)	1749 (12.3)	1739 (12.3)	1494 (11.5)	
Moderate exercise (n = 73,605)							< 0.001
None or ≤ 15 min	21,121 (28.7)	3548 (23.3)	4009 (26.7)	4208 (28.3)	4538 (30.7)	4818 (35.2)	
16–29 min	24,712(33.6)	4976(32.7)	5117 (34.0)	5158 (34.7)	5015 (33.9)	4446 (32.5)	
≥ 30 min	27,772(37.8)	6685 (43.9)	5910 (39.4)	5512 (37.0)	5236 (35.4)	4429 (32.3)	
Family history of lung cancer (%)							< 0.001
No	83,499 (87.1)	16,992 (87.2)	16,804 (87.1)	16,939 (87.6)	16,927 (87.3)	15,837 (86.2)	
Yes, family members	10,058 (10.5)	2060 (10.6)	2038 (10.6)	1973 (10.2)	1976 (10.2)	2011 (10.9)	
Yes, relatives or unclear cancer types	2311 (2.4)	431 (2.2)	444 (2.3)	432 (2.2)	480 (2.5)	524 (2.9)	
Chronic bronchitis = yes (%)	4070 (4.2)	807 (4.1)	810 (4.2)	793 (4.1)	840 (4.3)	820 (4.4)	0.413
Colon comorbidity = yes (%)	1279 (1.3)	233 (1.2)	251 (1.3)	251 (1.3)	255 (1.3)	289 (1.6)	0.026
Diabetes = yes (%)	6327 (6.6)	1212 (6.2)	1196 (6.2)	1195 (6.2)	1284 (6.6)	1440 (7.8)	< 0.001
Diverticulitis = yes (%)	6539 (6.8)	1399 (7.2)	1369 (7.1)	1307 (6.7)	1347 (6.9)	1117 (6.1)	< 0.001
Emphysema = yes (%)	1983 (2.1)	325 (1.7)	343 (1.8)	402 (2.1)	475 (2.4)	438 (2.4)	< 0.001
Heart attack = yes (%)	7968 (8.3)	1415 (7.3)	1606 (8.3)	1677 (8.6)	1626 (8.4)	1644 (8.9)	< 0.001
Hypertension = yes (%)	31,174 (32.4)	5869 (30.1)	6125 (31.7)	6359 (32.8)	6443 (33.1)	6378 (34.6)	< 0.001
Liver comorbidity = yes (%)	3461 (3.6)	752 (3.9)	654 (3.4)	662 (3.4)	696 (3.6)	697 (3.8)	0.039
Intestinal polyps = yes (%)	6415 (6.7)	1255 (6.4)	1354 (7.0)	1327 (6.8)	1357 (7.0)	1122 (6.1)	0.001
Stroke = Yes (%)	1916 (2.0)	365 (1.9)	372 (1.9)	377 (1.9)	387 (2.0)	415 (2.3)	0.073
Total energy (kcal/day), mean (SD)	1860.9(709.8)	1729.9 (653.0)	1845.1 (683.0)	1890.5(703.0)	1922.7 (727.3)	1920.0 (762.8)	< 0.001
Alcohol intake (gm/day), median [IQR]	1.6 [0.0, 8.9]	1.5 [0.0, 9.6]	2.0 [0.3, 10.4]	2.0 [0.2, 10.3]	1.6 [0.0, 8.4]	1.1 [0.0, 6.2]	< 0.001
Carbohydrate (gm/day), median [IQR]	204.5 [157.0, 261.6]	199.1 [154.2, 256.6]	205.6 [158.5, 261.3]	206.4 [158.8, 261.0]	207.1 [160.2, 263.7]	204.1 [152.9, 265.5]	< 0.001
Protein (gm/day), median [IQR]	60.4 [45.3,79.4]	61.0 [46.2, 79.9]	62.3 [46.8, 81.5]	61.5 [46.5, 80.9]	61.3 [45.7, 80.8]	55.7 [41.4, 73.5]	< 0.001
Fat (gm/day), median [IQR]	54.5 [38.6,75.7]	47.2 [33.7, 65.6]	53.8 [38.4, 74.5]	57.3 [40.7, 78.6]	58.9 [41.8, 81.1]	56.3 [39.8, 78.2]	< 0.001
Cholesterol (mg/day), median [IQR]	173.5 [116.3, 255.5]	147.9 [98.5, 219.9]	171.4 [116.8, 251.2]	183.5 [124.0, 266.4]	188.7 [126.4, 274.3]	178.7 [119.5, 262.9]	< 0.001
Saturated fatty acid (gm/day), median [IQR]	16.9 [11.7, 24.2]	13.7 [9.6, 19.5]	16.3 [11.4, 23.1]	17.8 [12.5, 25.1]	19.0 [13.2, 26.6]	18.5 [12.7, 26.3]	< 0.001
MUFA (gm/day), median [IQR]	20.4 [14.2,28.7]	17.5 [12.3, 24.8]	20.2 [14.2, 28.3]	21.5 [15.0, 29.8]	22.1 [15.4, 30.8]	21.2 [14.8, 29.8]	< 0.001
PUFA (gm/day), median [IQR]	12.3 [8.7,17.1]	11.5 [8.1, 16.1]	12.5 [8.9, 17.4]	12.9 [9.2,17.8]	12.8 [9.1, 17.6]	11.9 [8.3, 16.6]	< 0.001
Trans-fatty (gm/day), median [IQR]	3.4 [2.3, 4.9]	2.7 [1.9, 3.9]	3.3 [2.3, 4.7]	3.6 [2.5, 5.2]	3.8 [2.6, 5.4]	3.7 [2.5, 5.3]	< 0.001
Proportion of UPF, % gm/day							
Mean (SD)	31.2 (14.0)	13.4 (4.0)	22.8 (2.2)	30.2 (2.2)	38.3 (2.6)	52.7 (8.2)	< 0.001
95% CI	31.14, 31.32	13.30,13.41	22.75, 22.82	30.17, 30.23	38.25, 38.32	52.55, 52.78	
Proportion of UPF, % kcal							
Mean (SD)	37.1 (11.2)	21.8 (4.6)	31.05 (1.8)	36.85 (1.58)	42.81 (1.94)	53.17 (6.032)	< 0.001
95% CI	37.06, 37.21	21.79, 21.93	31.03, 31.08	36.82, 36.87	42.78, 42.84	53.09, 53.26	
HEI-2015 score, mean (SD)	66.8 (9.6)	72.6 (8.1)	69.2 (8.4)	66.8 (8.7)	64.6 (8.8)	60.4 (9.4)	< 0.001

*CI* confidence interval, *HEI* Healthy Eating Index, *IQR* Inter-quartile range, *MUFA* monounsaturated fatty acid, *PUFA* polyunsaturated fatty acid, *SD* standard deviation

^a^Chi-Square (X^2^) for categorical variables and analysis of variance for continuous variables; Other includes unemployed, disabled, and extended sick leave

A higher proportion of participants in the highest intake quintile compared to the lowest quintile had higher total energy intake, spent less time on physical activity, had a lower socioeconomic status, a lower healthy eating index (HEI) score (UPF intake negatively correlated with HEI-2015, with a correlation coefficient r = − 0.445), and higher carbohydrate, fat, saturated fat, cholesterol, and trans-fatty acid consumption (Table 1). Consumption of cookies and pies, milk desserts, processed meat and sausage, sugary drinks and sweet products, potato salad, and ready-to-eat salty snacks were much higher among participants in the highest quintile of UPF consumption compared to the lowest (Supplementary Fig. 2).

### UPF consumption and mortality from overall respiratory, lung cancer, and COPD

During the 1,379,655.5 person-years follow-up time (median [IQR] 16.8 [11.9–18.6] years), there were 28,700 all-cause, 4,092 all respiratory diseases, 2015 lung cancer and 1536 COPD related mortalities. Participants in the highest quintiles of UPF consumption had a 10% higher risk of overall CRD mortality (HR 1.10; 95% CI 1.01, 1.21), 26% higher risk mortality due to COPD (HR 1.26; 95% CI 1.06, 1.49) and 18% higher all-cause mortality risk (HR 1.18; 95% CI 1.13, 1.22) compared to participants in the first quintile. In the unadjusted and age-sex adjusted model, a higher proportion of UPF consumption was associated with a higher risk of lung cancer mortality but not in the fully adjusted model (HR 0.97; 95% CI 0.84, 1.12).

### Dose–response analysis

**Fig. 1:**
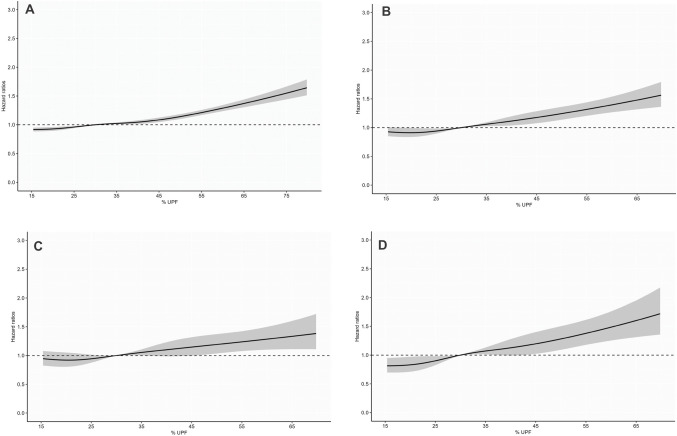
Restricted cubic spline analysis of the association UPF consumption and **A** all-cause mortality, **B** overall CRD mortality, **C** lung cancer mortality and **D** COPD mortality. The shaded areas below and above the smooth solid line are the 95% confidence interval for the fitted line

A 5% absolute increase in the proportion of UPF consumption in the total diet was associated with an increased risk of all-cause mortality, overall CRD mortality, COPD mortality and lung cancer mortality by 9% (HR 1.09; 95% CI 1.06, 1.13; *p-nonlinear* < *0.001*), 12% (HR 1.12; 95% CI 1.03,1.21; *p-nonlinear* < *0.001*), 17% (HR 1.17; 95% CI 1.02, 1.35; *p-nonlinear* = *0.013*) and 8% (HR 1.08; 95% CI 0.95, 1.22; *p-nonlinear* = *0.41*) respectively (Table 2**, **Fig. 1).

**Table 2:** Cox proportional model for the association between UPF consumption, lung cancer, COPD and overall CRDS mortality in older adults enrolled in PLCO Trial, USA

	Quintiles of proportion of UPF in the total diet (% weight)								Per 5% increment in UPF intake HR (95% CI)	*P* non-linear
	Q1(lowest)	Q2		Q3		Q4	Q5(highest)	*P* for trend		
	Ref	HR (95% CI)		HR (95% CI)		HR (95% CI)	HR (95% CI)			
All-cause mortality										
Number of participants	18,190	18,371		18,572		18,650	17,670			
Follow-up time, median [IQR]	17.1 [12.3, 18.7]	17.0 [12.1, 18.7]		16.9 [11.8, 18.6]		16.7 [11.7, 18.6]	16.6 [11.6, 18.5]			
Person-years	280,053.6	280,467.8		279,517.0		278,506.5	261,110.6			
Number of deaths	5260	5560		5954		6064	5862			
Mortality rate /1000	18.8	19.8		21.3		21.7	22.4			
Unadjusted model	Ref	1.09 (1.04, 1.14)		1.16 (1.11, 1.22)		1.28 (1.22,1.34)	1.45 (1.38,1.52)	< 0.0001	1.13 (1.09,1.17)	0.002
Age-sex adjusted model^a^	Ref	1.03 (0.99,1.07)		1.09 (1.06, 1.14)		1.15 (1.10,1.19)	1.33 (1.28, 1.38)	< 0.0001	1.13 (1.10, 1.17)	< 0.001
Multivariable adjusted model^b^	Ref	1.0 (0.96, 1.04)		1.06 (1.02,1.10)		1.07 (1.03,1.11)	1.18 (1.13, 1.22)	< 0.0001	1.09 (1.06, 1.13)	0.001
Overall CRDs mortality										
Number of deaths	733	743		820		903	893			
Mortality rate/10,000	26.0	26.3		29.2		32.3	34.0			
Unadjusted model	Ref	1.01 (0.92,1.12)		1.12 (1.02,1.24)		1.25 (1.14,1.38)	1.33 (1.20,1.46)	< 0.0001	1.27 (1.17, 1.38)	0.140
Age-sex adjusted model^a^	Ref	0.97 (0.88,1.08)		1.06 (0.96,1.17)		1.18 (1.07,1.31)	1.35 (1.23, 1.50)	< 0.0001	1.25 (1.15, 1.36)	0.002
Multivariable adjusted model^b^	Ref	0.93 (0.84, 1.03)		0.98 (0.89,1.09)		1.02 (0.93,1.13)	1.10 (1.01, 1.22)	0.009	1.12 (1.03, 1.22)	0.01
Lung cancer mortality										
Number of deaths	376	366		403		439	431			
Mortality rate /10,000	13.0	13.0		14.0		16.0	16.0			
Unadjusted model		0.98 (0.85,1.132)		1.09 (0.95,1.26)		1.22 (1.06,1.40)	1.35 (1.18, 1.55)	< 0.0001	1.21 (1.07, 1.37)	0.217
Age and sex adjusted model^a^	Ref	0.93 (0.80,1.07)		1.00 (0.87, 1.15)		1.09 (0.95,1.25)	1.21 (1.05, 1.39)	0.0006	1.17 (1.03, 1.32)	0.048
Multivariable adjusted model^b^	Ref	0.88 (0.76, 1.02)		0.93 (0.81,1.07)		0.96 (0.83,1.11)	0.97 (0.84, 1.12)	0.79	1.08 (0.95,1.22)	0.224
COPD mortality										
Number of deaths	258	278		312		348	340			
Mortality rate/10,000	9.2	9.9		11.2		12.5	13.0			
Unadjusted model	Ref	1.10 (0.93, 1.30)		1.27 (1.07, 1.49)		1.47 (1.25,1.72)	1.71 (1.44,2.00)	< 0.000	1.34 (1.16, 1.54)	0.302
Age and sex adjusted model^a^	Ref	1.05 (0.89,1.24)		1.18 (1.00,1.39)		1.35 (1.15,1.59)	1.58 (1.34,1.87)	< 0.000	1.35 (1.17, 1.55)	0.532
Multivariable adjusted model^b^	Ref	0.98 (0.83,1.16)		1.05 (0.89,1.24)		1.07 (0.90,1.26)	1.26 (1.06, 1.49)	0.004	1.17 (1.02,1.35)	0.650

*COPD* chronic obstructive pulmonary disease, *CRDs* chronic respiratory diseases, *HR* hazard ratio

^a^Model adjusted for age (timescale variable) and sex

^b^Model adjusted age, sex, marital status, education, race, study arm, occupation, history of lung cancer, hypertension, diabetes, stroke, heart attack, liver comorbidity, colon comorbidity, aspirin and ibuprofen use, cigarette smoking, alcohol intake (gm/day), BMI, emphysema, chronic bronchitis, and total dietary energy

### Subgroup analysis

In the subgroup analysis, the magnitude of UPF consumption and risk of mortality from overall respiratory, COPD and lung cancer did not vary across subgroups when stratified by sex, age, BMI, smoking, and hypertension, diabetes, chronic bronchitis, and emphysema (all p-interaction > 0.05). However, the risk of mortality from COPD differed by baseline BMI (HR 0.81; 95% CI 0.56, 1.16 for normal, HR 0.69; 95% CI 0.48, 0.99 for overweight, HR 1.01; 95% CI 0.72, 1.41 for obesity) and chronic bronchitis status (HR 0.65; 95% CI 0.46, 0.93 for no bronchitis vs HR 0.90; 95% CI 0.63, 1.27 for people with bronchitis) and both BMI and bronchitis had statistically significant interactions with COPD mortality (both *p-for interaction* < *0.05*) (Table 3).

**Table 3:** Subgroup analysis on the association between UPF consumption and lung cancer, COPD, and overall CRDs mortality among older adults from PLCO trial USA

Subgroup variables	Overall CRDs mortality		COPD mortality		Lung cancer mortality		All-cause mortality	
	HR_quintile 5 vs 1_ (95% CI)^a^	*P*_interactions_	HR_quintile 5 vs 1_ (95% CI)^a^	*P*_interactions_	HR_quintile 5 vs 1_ (95% CI)^a^	*P*_interactions_	HR_quintile 5 vs 1_ (95% CI)^a^	*P*_interactions_
Sex								
Male	1.13 (0.99, 1.30)	0.67	1.11 (0.88, 1.41)	0.75	0.85 (0.68, 1.06)	0.30	1.11 (1.05, 1.17)***	0.54
Female	1.09 (0.94, 1.27)		1.37 (1.08, 1.74)**		0.94 (0.76, 1.16)		1.24 (1.17, 1.31)***	
Age (years)								
55–59	1.51 (1.06, 2.15)*	0.18	1.00 (0.77, 1.28)	0.10	0.95 (0.75, 1.19)	0.52	1.03 (0.98, 1.09)	0.26
60–64	1.01 (0.81, 1.24)		1.14 (0.89, 1.46)		0.93 (0.74, 1.16)		1.09 (1.04, 1.15)***	
65–69	1.24 (1.04, 1.49)*		1.25 (0.98, 1.59)		0.91 (0.72, 1.14)		1.10 (1.04, 1.16)***	
≥ 70	1.25 (1.07, 1.47)**		1.41 (1.10, 1.80)**		0.97 (0.76, 1.23)		1.18 (1.11, 1.25)***	
Cigarette smoking								
Never smoker	1.09 (0.84, 1.41)	0.92	1.07 (0.86, 1.35)	0.56	1.02 (0.84, 1.23)	0.23	1.11 (1.06, 1.18)***	0.06
Current smoker	1.24 (1.04, 1.48)*		1.15 (0.92, 1.43)		1.12 (0.93, 1.35)		1.13 (1.07, 1.20)***	
Former smoker	1.21 (1.05, 1.39)**		1.28 (1.02, 1.61)*		1.04 (0.85, 1.26)		1.25 (1.19, 1.32)***	
BMI (kg/m^2^)								
< 18.5	2.08 (0.87, 4.96)	0.08	0.83 (0.57, 1.18)	0.0009	0.80 (0.57, 1.12)	0.77	0.97 (0.89, 1.05)	0.54
18.5–24.99	1.26 (1.07, 1.49)**		0.81 (0.56, 1.16)		0.84 (0.61, 1.17)		1.08 (0.99, 1.16)	
25–29.99	1.15 (0.97, 1.34)		0.69 (0.48, 0.99)*		0.85 (0.62, 1.17)		1.06 (0.98, 1.14)	
≥ 30	1.10 (0.89, 1.35)		1.01 (0.72, 1.41)		0.92 (0.67, 1.25)		1.19 (1.10, 1.28)***	
Diabetes history								
No	1.19 (1.07, 1.32)***	0.17	1.07 (0.61, 1.89)	0.85	0.87 (0.49, 1.56)	0.06	1.07 (0.96, 1.19)	0.56
Yes	1.37 (1.01, 1.86)*		0.94 (0.51, 1.73)		1.54 (0.92, 2.59)		1.27 (1.14, 1.41)***	
Hypertension history								
No	1.20 (1.06, 1.36)**	0.57	0.94 (0.72, 1.23)	0.59	0.96 (0.75, 1.23)	0.97	1.07 (1.01, 1.13)*	0.16
Yes	1.20 (1.02, 1.42)*		1.19 (0.92, 1.55)		1.04 (0.81, 1.32)		1.22 (1.14, 1.29)***	
Emphysema history								
No	1.18 (1.05, 1.31)**	0.24	0.79 (0.57, 1.10)	0.23	0.82 (0.48, 1.39)	0.22	0.88 (0.73, 1.04)	0.07
Yes	1.35 (1.02, 1.79)*		1.10 (0.79, 1.52)		0.96 (0.57, 1.62)		0.98 (0.81, 1.17)	
Bronchitis history								
No	1.21 (1.09, 1.35)***	0.55	0.65 (0.46, 0.93)*	0.02	1.43 (0.83, 2.42)	0.33	1.01 (0.87, 1.18)	0.72
Yes	1.19 (0.89, 1.59)		0.90 (0.63, 1.27)		1.05 (0.59, 1.86)		1.14 (0.98, 1.33)	
Study arm								
Intervention	1.23 (1.07, 1.42)**	0.46	1.37 (1.09, 1.73)**	0.08	1.11 (0.92, 1.34)	0.96	1.36 (1.29, 1.43)***	0.23
Control	1.17 (1.01, 1.35)*		1.30 (1.03, 1.65)*		1.00 (0.81, 1.23)		1.29 (1.21, 1.35)***	

*COPD* chronic obstructive pulmonary diseases, *CRDs* chronic respiratory diseases, *HR* hazard ratio

*Significant at p-value < 0.05

**Significant at p-value < 0.01

***Significant at p-value < 0.001

^a^Adjusted for age in years, sex, race, education, marital status, occupation, study arm, history of lung cancer, hypertension, diabetes, stroke, emphysema, bronchitis, smoking, liver comorbidity, arthritis, colon comorbidity, aspirin, Ibuprofen, BMI, alcohol drinking (gm/day), and total energy (kcal/day)

A positive association between UPF consumption and risk of all-cause mortality (HR 1.24; 95% CI 1.17, 1.31), and COPD mortality (HR 1.37; 95% CI 1.08, 1.74) were more pronounced among females than males (*p-interaction for all* > *0.05*). The effect of UPF consumption on overall CRD mortality was higher among current (HR 1.24; 95% CI 1.04, 1.48) and former smokers (HR 1.21; 95% CI 1.05, 1.39) and those with diabetes (HR 1.37; 95% CI 1.01, 1.86) compared to those who never smoked or did not have diabetes (HR 1.09; 95% CI 0.84,1.41 for never smokers and HR 1.19; 95% CI 1.07,1.32 for those without diabetes and both *p-for interaction* > *0.05*) (Table 3).

The cumulative incidence of mortality from overall respiratory diseases, lung cancer and COPD was compared with competing events over the fifth, 10th and 20th years of follow-up, grouped by the quintiles of UPF consumption (Supplementary Table 3, Supplementary Fig. 3). Competing risk regression analysis demonstrated that participants in the highest quintile of UPF consumption had a higher risk of overall CRDs mortality (sub-distributional HR 1.07; 95% CI 1.01, 1.19) and mortality from COPD (sub-distributional HR 1.20; 95% CI 1.02, 1.42) compared to participants in the lowest quintile (Table 2, Supplementary Table 3).

### Sensitivity analysis

Sensitivity analysis showed that the estimates of the association between UPF consumption and mortality from overall CRD and COPD were consistent with results obtained from the main fully adjusted Cox regression models. After MI, the risk of mortality from CRDs overall, COPD and lung cancer increased among participants in the highest quintile of UPF consumption (HR 1.26; 95% CI 1.16, 1.39; HR 1.45; 95% CI 1.22, 1.70; HR 1.16; 95% CI 1.01, 1.34) compared to the lowest quintile, respectively. The lung cancer results obtained from imputed data differed from the complete cases analysis (Supplementary Table 4).

Mortality risks were higher for participants who consumed higher amounts of UPF (gm/day) for CRDs (HR 1.14; 95% CI 1.04, 1.24), COPD (HR 1.18; 95% CI 1.01, 1.38), and lung cancer (HR 1.02; 95% CI 0.89, 1.17). However, when taking into account the consumption of artificial and sugary beverages, these risks were significantly reduced. There were no noticeable changes when considering other subgroups of ultra-processed foods intake or the intake of unprocessed or minimally processed foods (such as fish, fruits, and vegetables) (Supplementary Table 5).

Furthermore, after taking into account the intensity of cigarette smoking in the final imputed model, it was observed that individuals in the highest quintile of UPF consumption had significantly elevated risks of mortality from CRDs (HR 1.34; 95% CI 1.22, 1.47), COPD (HR 1.57; 95% CI 1.33, 1.85), and lung cancer (HR 1.22; 95% CI 1.06, 1.40) compared to those in the lowest quintile (Supplementary Table 6).The final Cox regression analysis also suggested significant positive associations between UPF consumption in total calories and mortality due to overall CRDs (HR 1.30; 95% CI 1.17, 1.43), COPD (HR 1.53; 95% CI 1.29,1.81) and lung cancer (HR 1.19 (1.03, 1.37) (Supplementary Table 7).

## Discussion

The findings of this study showed that the risk of mortality from CRDs and COPD increased by 10% and 26% respectively among participants in the highest quintile of UPF intake compared to the lowest quintile. A 5% increase in the proportion of UPF consumption was associated with a 12% and 17% increase in the relative hazards of overall CRD and COPD mortality, respectively. After accounting for the effect of competing events, this study confirmed that consumption of UPF increased the risk of mortality from overall CRDs by 7% and COPD by 20% but there was no evidence of association with lung cancer mortality. However, in the final imputed models, participants with higher consumption of UPF (% gm/day) had a significantly increased risk of lung cancer mortality by 16%. These inconclusive findings from complete-case and MI analyses could be due to the theoretical assumption. We believe that missingness is unrelated to the risk of outcomes in this study, as covariates were measured at baseline before the events occurred. In this case, MI leads to bias away from the null, while complete-case analysis has minimal bias. Furthermore, the standard errors in the complete-case analyses of the current study are smaller compared to MI analyses. Considering these factors, estimates from complete cases may be less biased than MI [39].

Another noteworthy finding of the study was that females in the highest quintile of UPF consumption compared to the lowest quintile had a disproportionately higher risk of COPD mortality (37%). Moreover, higher UPF intake among participants aged 70 years and over and ex-smokers was significantly associated with a higher risk of COPD mortality (41% and 28% respectively). In the subgroup analyses, it was found that the risk of mortality from CRDs overall, COPD, and lung cancer associated with UPF consumption was lower among non-smokers compared to current and former smokers. It is important to note that this difference was observed even in the absence of a significant interaction. This could be because among non-smokers, the overall lifestyle is healthier, and the number of mortality cases is small. For example, in this study, the mean HEI-2015 score was higher in non-smokers (68, 95% CI 67.69–67.87) than in current smokers (61, 95% CI 60.91–61.35), and there were fewer deaths from CRDs, COPD, and lung cancer in non-smokers compared to current smokers: 894, 187, 179 vs 1,464, 572, and 763, respectively. Moreover, participants in the highest quintile of UPF intake (mean = 60) had a lower mean HEI-2015 score than participants in the lowest quintile (mean = 73).

In our study, UPF intake accounted for about 31% of the total diet (% weight/day) and 37% of the total calories among older adults (≥ 55 years) in the USA. However, in the general population of the USA, UPF makes up more than 50% [41] of the total energy intake. Among the younger population, this contribution reaches] about two-thirds [42]. The current the current study also revealed notable differences in UPF consumption patterns among participants with lower family income, younger adults, overweight and people with obesity, as well as non-smokers and ex-smokers. This study aligns with previous research [41, 43, 44] by demonstrating that UPF consumption is inversely an inverse related to dietary quality as measured by HEI-2015, lower intake of fruits and vegetables, and positively related to total fat, cholesterol, saturated fat, and trans-fatty acid intake.

### Comparison with other studies

To our knowledge no studies have identified the association between the proportion of UPF consumption in the total diet (% gm/day) and mortality due to overall CRDs, COPD and lung cancer in the USA. However, there are a number of studies that have demonstrated positive associations between UPF consumption all-cause mortality [14, 29, 45–49], cardiovascular mortality [17, 46–51], depression and other mental health problem [49, 51–53] and some cancer types [31, 54, 55].

A recent study from the UK Biobank suggested that a higher UPF intake increases the risk of respiratory diseases/incidence by 4% but the risk of respiratory mortality increased by 12%, a non-statistically significant association. Two studies in Brazil (one cross-sectional study with small samples (n = 513) and one national survey) showed a positive relationship of UPF consumption and respiratory diseases (asthma and wheezing) in adolescents and risk of wheezing in children [19, 20]. In line with these findings, the current study revealed higher UPF intake is responsible for at least a 10% increase in the risk of overall CRDs mortality and a 26% higher risk of COPD mortality in older adults in the USA. The discrepancy between the current study and that using the UK Biobank data may be due to a shorter follow-up period (10 years) and lower number of respiratory-related deaths (n = 593) of the previous one. In addition, another study from UK Biobank estimated a 38% higher risk of lung cancer mortality among individuals with highest UPF intakes [30] but a study from 10 European countries showed a non-statistically significant association between UPF and lung cancer mortality [49]. The current study supports this result by indicating a non-statistically significant higher risk of lung cancer mortality, using Cox and competing risk regression analyses of complete cases, among participants with the highest UPF intake.

### Potential mechanisms

Several theories related to the association between UPF consumption and risk of mortality from the underlying causes of respiratory diseases have been proposed. Firstly, consumption of UPF is the predominant dietary pattern, displacing healthier foods in the American diet and globally. UPF are nutritionally inferior, poor sources of essential nutrients such as antioxidants and rich in energy, sugary drinks, saturated and hydrogenated fats [43, 44]. These products all cause systemic oxidative stress and breach immunological response [50], which could be the prime mechanism for the development of many non-communicable diseases [56, 57], including CRDs and cancer. Epidemiological studies show that dietary antioxidants are paramount for respiratory epithelial cell integration and pulmonary function in antioxidant gene polymorphic forms [58, 59]. One experimental study showed that dietary antioxidant formulation has a potential to act as a chemo-protectant by lowering reactive oxygen species and DNA damage caused by gamma-radiation in human bronchial epithelial cell. This reduces the risk of chronic inflammatory diseases and lung cancer [60].

Other mechanisms may be linked to industrial processing and packaging of UPF that involve alteration in the structure of food matrices [57], additives and neoformed contaminants which adversely affect human health [61]. Heat treatment or thermal processing of foods (from baking, coffee, fruit, meat, milk, fish, sugars and alcohol) are potential sources of toxic chemicals including acrylamide, furan, furfural, nitrosamines, heterocyclic amines and acrolein which are classified as carcinogenic to humans by the International Agency for Research on Cancer [62, 63]. Studies have revealed that aspartame, is an artificial sweetener associated with an increased risk of overall cancer [64, 65] and food additives (preservatives, antioxidants, and flavour enhancers) cause hypersensitivity reactions in the respiratory tract[66]. Reaction of nitrite and nitrate with secondary amines from processed meat generates N-nitrosamines, are also carcinogenic to humans [62, 67]. Additionally, UPF is a potential source of the most used additive, titanium dioxide, and studies have shown a risk of chronic inflammation and carcinogenesis, including lung tumours associated with the use of titanium dioxide [9, 62]. Furthermore, dietary emulsifiers have also been implicated in promoting gut inflammation and alteration in microbiota which lead to an increased risk of cancer [68]. However, findings in this area are inconclusive [68–71]

Finally, with regard to the packaging of UPF. This could contain endocrine-disrupting chemicals such as bisphenol A and phthalate which may impose negative health effect including cancer and hormonal disruptions [71–73].

### Implications

Despite of all of the available evidence, there has not been a strong focus on implementing unified and impactfully policies and regulation to reduce high consumption of UPF and promoting healthy diets. While more than 45 countries have created taxes on sugar-sweetened beverages, only a few have approved taxes on snacks and other UPF, and none have created major subsidies for healthy unprocessed or minimally processed foods for those of lower socioeconomic status [74]. Public health efforts should be invested in evaluating the wider impact of UPF consumption (economic impact and inequalities, political, sociocultural, behavioural factors, and the planetary welfare in general) and structural interventions aimed at increasing access to convenient, and affordable minimally processed foods [75].

### Strengths and limitations of the study

The use of a large sample size, long follow-up period and validated FFQ are all strengths of this study. However, there are limitations. The PLCO trial participants were relatively older adults (55–74 years) and thus the findings of this study may have limited generalizability. Secondly, the dietary data are self-reported and could be prone to recall bias and was initially gathered approximately 22 years ago. Given this substantial time gap, changes in dietary consumption patterns over the years could potentially impact the precise estimation of the association between UPF intake and mortality resulting from overall CRDs, COPD, and lung cancer. The proportion and energy share of UPF are notably lower in our study compared to recent findings in America. Despite this, the validated FFQ that we used has been shown to be reliable in recording the usual dietary intake of participants. Thus, even though some individuals' dietary consumption may have significantly changed over time, this shift might not significantly affect the observed associations. Due to lack of uniform techniques in classifying food items to the NOVA food group 4, misclassification bias might be introduced but extensive effort was made to use the standard definition of UPF and referenced previously published materials to provide consistency [28, 36, 76]. Even though we adjusted the model with a range of confounders, unmeasured/residual confounders (such as biological biomarkers, non-nutritional byproducts, additives and in-and out-door particulate matters) may affect the observed associations between UPF intake and mortality due respiratory diseases. The E-value for CRD overall was 1.43 on the risk ratio scale in this study. Thus, the observed hazard ratio of 1.10 could be explained away by unmeasured confounder that was associated with both UPF consumption and overall CRDs mortality by a risk ratio of 1.43-fold each, above and beyond the measured confounders but weaker confounding could not do so [29, 31]. Similarly, the E-value for COPD mortality was 1.83 with lower confidence interval of 1.32 which hypothetically indicates that unmeasured confounder explains away the association with both UPF and COPD mortality by 1.83-fold each, conditional on the measured covariates.

## Conclusions

The present study highlights the potential risk increase for all-cause mortality, overall CRDs, COPD, and lung cancer associated with increased consumption of UPF among older adults in the USA. It is important to corroborate these findings by conducting further research to replicate these analyses and focus on the biological and immunological mechanisms underlying UPF consumption. The investigation should encompass non-nutritional factors such as food additives, neoformed contaminants, and alterations in food structure related to UPF consumption and their potential risks for CRDs in the broader population. The increasing production and global dominance of UPF significantly augment the burden of NCDs, including cancer. As a primary intervention for NCDs, it is imperative to enact policies aimed at reformulating food products, taxing UPF, implementing impactful food labelling, and restricting the market for UPF. Simultaneously, the promotion of healthy, minimally processed foods for all individuals is critically important [75].

### Supplementary Information

Below is the link to the electronic supplementary material.

Supplementary file1 (DOCX 353 KB)

## Data Availability

The PLCO trial data are available upon request to The National Cancer Institute at Access to PLCO Data, Images, and Biospecimens—Learn—PLCO—The Cancer Data Access System.

## Abbreviations

BMI: Body Mass Index
COPD: Chronic obstructive pulmonary disease
CRDs: Chronic respiratory diseases
CRR: Computing risk regression
CVD: Cardiovascular disease
DHQ: Dietary History Questionnaire
FFQ: Food Frequency Questionnaire
HEI: Healthy Eating Index
HR: Hazard ratio
MI: Multiple imputation
NCDs: Non-communicable diseases
PLCO: Prostate, lung, colorectal and ovarian cancer
UPF: Ultra-processed food
