# Supplementary material for: Ultra-processed food consumption and risk of chronic respiratory diseases mortality among adults: evidence from a prospective cohort study
Source: Eur J Nutr. 2024 Feb 28;63(4):1357–72. doi: 10.1007/s00394-024-03356-4 (PMC11139728; doi:10.1007/s00394-024-03356-4)
Supplement: Supplementary file 1 — Supplementary file1 (DOCX 353 KB) [file 394_2024_3356_MOESM1_ESM.docx]

Ultra-Processed Food Consumption and Risk of Mortality from Overall Respiratory Diseases, Chronic Obstructive Pulmonary Disease and Lung Cancer: Evidence from a Prospective Study

Tefera Chane Mekonnen, Yohannes Adama Melaku, Zumin Shi, Tiffany K Gill

**Definition and Categorization of Ultra-Processed Food (UPF)**

**UPF (NOVA Group 4):** Food substances **c**ome from the subsequent processing of food components, such as hydrogenated oils, hydrolysed proteins, soy protein isolates, maltodextrin, invert sugar and high fructose corn syrup. They contain additives dyes, flavourings, flavour enhancers, artificial sweeteners, and processing aids such as thickeners, bulking agents, anti-foaming agents, anti-caking agents, emulsifiers, sequestering agents, and humectants. Food items included in this group are fruit drinks containing additives (colours, flavours, artificial sweeteners); industrial cheese or spreadable cheese which contain cream, or salt; yogurts containing sugar, flavour, colours, sweeteners, or thickener; sweetened breakfast cereals come from extrusion of wheat or corn; flavoured fruit desserts with added sugar, texturizing agents and colorants; fried seasoned vegetables; marinated in industrial sauces with added flavouring; and smoked or cured meats with added nitrites and conservatives.

Ham, processed meat (cold cuts, sausage, hamburger), packaged bread, pate, foie-gras, ultra-processed cheese(creamy), potato Starch, Onion Powder, Dried Carrot, Palm Oil, Dried Chicken, dried black pepper, salty snacks, sauces and dressings, liquors, sugar sweetened beverages, juice boxes, milkshakes, cookies, chocolate cookies, muffins, doughnuts or other non-handmade pastries, churros, chocolates and candies, breakfast cereals, sweetened yoghurt sugared ‘fruit’ yoghurts, Petit Suisse, ice cream and margarine. The ingredients of UPFs are not used in the culinary preparations.

Generally, we categorised food items as UPF if they are carbonated drinks, sweet or savory packaged snacks; ice cream, chocolate, candies (confectionery); mass-produced packaged breads and buns; margarines and spreads; industrial cookies (biscuits), pastries, cakes, and cake mixes; breakfast ‘cereals’, ‘cereal’ and ‘energy’ bars; ‘energy’ drinks; flavoured milk drinks; cocoa drinks; sweet desserts made from fruit with added sugars, artificial flavours and texturizing agents; cooked seasoned vegetables with ready-made sauces; meat and chicken extracts and ‘instant’ sauces; ‘health’ and ‘slimming’ products such as powdered or ‘fortified’ meal and dish substitutes; ready to heat products including pre-prepared pies, pasta and pizza dishes; poultry and fish ‘nuggets’ and ‘sticks’, sausages, burgers, hot dogs, and other reconstituted meat products, and powdered and packaged ‘instant’ soups, noodles and desserts.

Supplementary Figures

Study subject selection


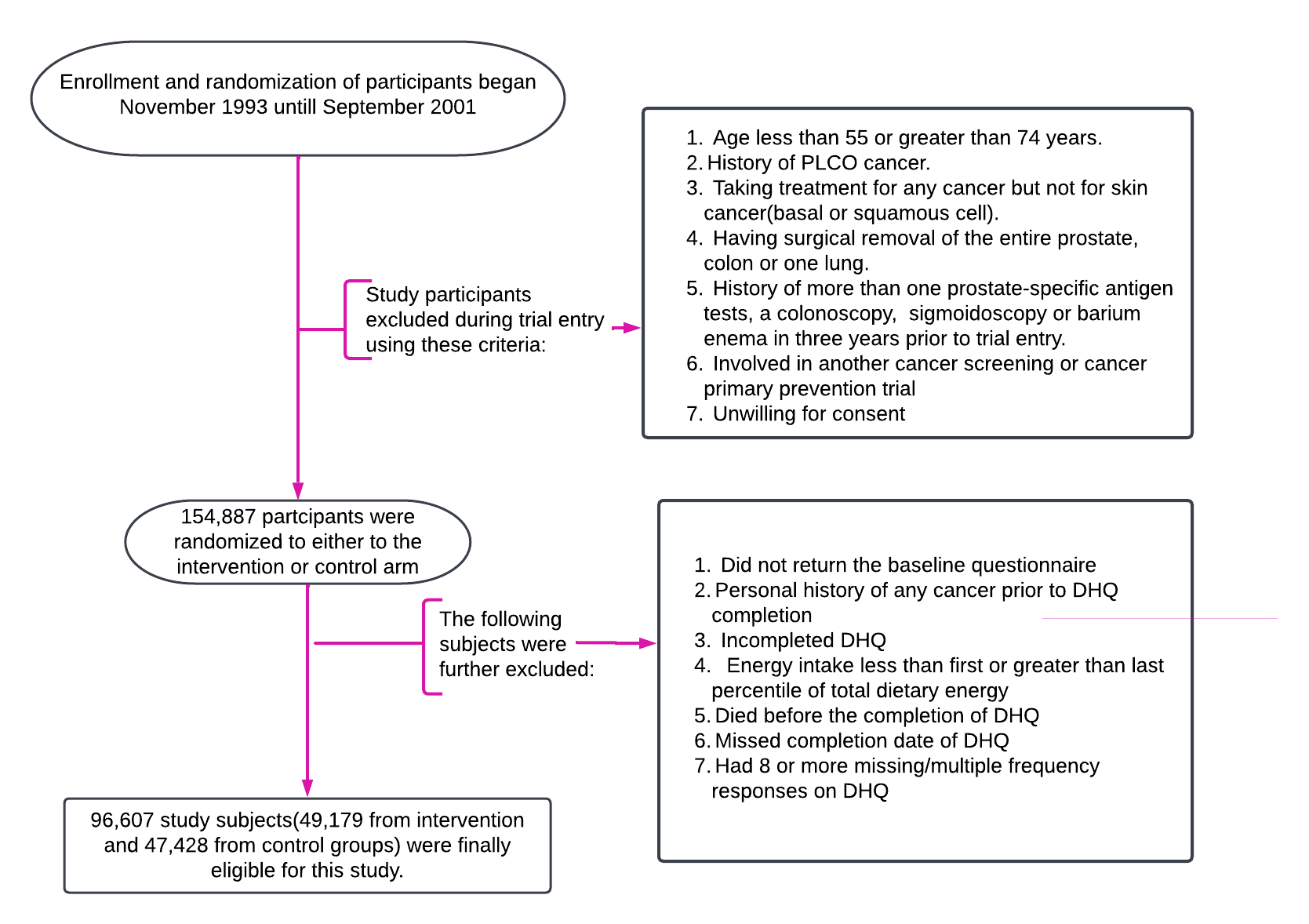


**Supplementary Figure 1**: Flowchart diagram showing the process of selecting eligible study subjects from Prostate, Lung, Colorectal and Ovarian cancer Trial (PLCO) in the United States of America^[[1]](#footnote-1)^.

**Supplementary Figure 2**: Distribution of median intakes(gm/day) of food subgroups by quintiles of proportion of UPF consumption in older adults of PLCO trial’s Participant in USA.

**Supplementary Tables**

**Supplementary Table 1:** Lists of food items belonging to the UPF category and corresponding energy estimated from the nutrition databases of USA.

| Sr. no | Food item code | Name of food item | Energy per 100gm (kcal) | Subgroups of UPF |
| --- | --- | --- | --- | --- |
|  | G_BISCUIT_DHQ | Biscuits | 370 | Cookies, and savoury foods |
|  | G_BREAD_WHITE_DHQ | White Bread/Rolls | 279 | Quick breads, ready-to-eat/heat grains |
|  | G_BUTTER_BRD_LOWFAT_DHQ | Butter, Reduced Fat on Bread | 662 | Salads, spreads, and sauces |
|  | G_BUTTER_BRD_REG_DHQ | Butter, Regular on Bread | 384 | Salads, spreads, and sauces |
|  | G_BUTTER_PAN_LOWFAT_DHQ | Butter, Reduced Fat on Pancakes/Waffles | 464 | Salads, spreads, and sauces |
|  | G_BUTTER_PAN_REG_DHQ | Butter, Regular on Pancakes/Waffles | 510 | Salads, spreads, and sauces |
|  | G_BUTTER_POT_LOWFAT_DHQ | Butter, Reduced Fat on Potatoes | 124 | Salads, spreads, and sauces |
|  | G_BUTTER_POT_REG_DHQ | Butter, Regular on Potatoes | 122 | Salads, spreads, and sauces |
|  | G_BUTTER_VEG_LOWFAT_DHQ | Butter, Reduced Fat on Vegetables | 82 | Salads, spreads, and sauces |
|  | G_BUTTER_VEG_REG_DHQ | Butter, Regular on Vegetables | 63 | Salads, spreads, and sauces |
|  | G_CAKE_LOWFAT_DHQ | Cakes, Low Fat | 283 | Cookies, and savoury foods |
|  | G_CAKE_REG_DHQ | Cakes, Regular | 389 | Cookies, and savoury foods |
|  | G_CANDY_CHOC_DHQ | Candy, Chocolate | 462 | Milk shakes, sweets, and condiments |
|  | G_CANDY_OTH_DHQ | Candy, Not Chocolate | 540 | Milk shakes, sweets, and condiments |
|  | G_CEREAL_OTHER_DHQ | Ready-to-Eat Cereal, Other | 382 | Quick breads, ready-to-eat/heat grains |
|  | G_CHEESECAKE_DHQ | Cheesecake | 321 | Cookies, and savoury foods |
|  | G_CHEESESCE_DHQ | Cheese Sauce | 160 | Salads, spreads, and sauces |
|  | G_CHIPS_LOWFAT_DHQ | Potato/Corn/Other Chips – Low Fat | 482 | Cookies, and savoury foods |
|  | G_CHIPS_OLESTRA_DHQ | Potato Chips with Olestra | 265 | Cookies, and savoury foods |
|  | G_CHIPS_REG_DHQ | Potato/Corn/Other Chips | 532 | Cookies, and savoury foods |
|  | G_CHOW_MEIN_DHQ | Chow Mein Noodles | 131 | Quick breads, ready-to-eat/heat grains |
|  | G_COBBLER_DHQ | Crisps/Cobblers | 157 | Cookies, and savoury foods |
|  | G_COFFEE_DECAF_NOSUG_DHQ | Coffee, Decaf - No Cream/No Sugar | 0 | Other UPF groups |
|  | G_COLDCUT_DHQ | Cold Cuts Excluding Ham | 208 | Animal based processed foods |
|  | G_COLDCUT_LOWFAT_DHQ | Cold Cuts, Low Fat | 208 | Animal based processed foods |
|  | G_COLDCUT_POULTRY_DHQ | Cold Cuts, Poultry | 98 | Animal based processed foods |
|  | G_COLDCUT_REG_DHQ | Cold Cuts, Regular | 188 | Animal based processed foods |
|  | G_COOKIE_LOWFAT_DHQ | Cookies and Brownies, Low Fat | 293 | Cookies, and savoury foods |
|  | G_COOKIE_REG_DHQ | Cookies and Brownies | 492 | Cookies, and savoury foods |
|  | **G_CORNBREAD_DHQ** | Corn Bread/Muffins | 305 | Quick breads, ready-to-eat/heat grains |
|  | G_COTTAGECH_DHQ | Cottage/Ricotta cheese | 84 | Milk shakes, sweets, and condiments |
|  | G_CRACKER_DHQ | Crackers | 510 | Quick breads, ready-to-eat/heat grains |
|  | G_CREAMCH_LOWFAT_DHQ | Cream Cheese, Low Fat | 231 | Milk shakes, sweets, and condiments |
|  | G_CREAMCH_REG_DHQ | Cream Cheese, Regular | 350 | Milk shakes, sweets, and condiments |
|  | G_CRM_COF_DHQ | Cream, Regular or Half & Half in Coffee and Tea | 136 | Milk shakes, sweets, and condiments |
|  | G_CRM_ND_LQD_DIET_DHQ | Non-Dairy Creamer, Liquid/Diet in Coffee, and Tea | 32 | Milk shakes, sweets, and condiments |
|  | G_CRM_ND_LQD_REG_DHQ | Non-Dairy Creamer, Liquid/Regular in Coffee, and Tea | 136 | Milk shakes, sweets, and condiments |
|  | G_CRM_ND_PWDR_DIET_DHQ | Non-Dairy Creamer, Powdered/Diet in Coffee, and Tea | 529 | Milk shakes, sweets, and condiments |
|  | G_CRM_ND_PWDR_REG_DHQ | Non-Dairy Creamer, Powdered/Regular in Coffee, and Tea | 482 | Milk shakes, sweets, and condiments |
|  | G_CRM_SOUR_LOWFAT_DHQ | Sour Cream, Low Fat | 74 | Milk shakes, sweets, and condiments |
|  | G_CRM_SOUR_REG_DHQ | Sour Cream, Regular | 198 | Milk shakes, sweets, and condiments |
|  | G_CRM_WHIPPED_REG_DHQ | Whipped Cream, Regular | 217 | Milk shakes, sweets, and condiments |
|  | G_CRM_WHIPPED_SUB_DHQ | Whipped Cream Substitute | 189 | Other UPF groups |
|  | G_CROISSANT_DHQ | Croissants | 406 | Cookies, and savoury foods |
|  | G_DONUT_DHQ | Donuts, Sweet Rolls, Danishes, and Pop Tarts | 403 | Cookies, and savoury foods |
|  | G_EGG_ROLLS_DHQ | Egg Rolls | 269 | Animal based processed foods |
|  | G_EGG_SUB_FA_DHQ | Egg Substitutes - Fat Added | 158 | Other UPF groups |
|  | G_EGG_SUB_NFA_DHQ | Egg Substitutes - No Fat Added | 84 | Other UPF groups |
|  | G_ENGL_MUF_BAGEL_DHQ | English Muffins and Bagels | 227 | Cookies, and savoury foods |
|  | G_FRUITPUNCH_DIET_DHQ | Fruit Drinks, Diet | 1 | Artificial and sugary-sweetened drinks |
|  | G_FRUITPUNCH_REG_DHQ | Fruit Drinks, Regular | 10 | Artificial and sugary-sweetened drinks |
|  | G_GELATIN_DHQ | Gelatins | 105 | Milk shakes, sweets, and condiments |
|  | G_GRANOLA_DHQ | Granola Bars | 471 | Quick breads, ready-to-eat/heat grains |
|  | G_HAM_LUNCH_DHQ | Ham, Cold Cuts or Lunch Meat | 188 | Animal based processed foods |
|  | G_HAM_LUNCH_LOWFAT_DHQ | Ham, Cold Cuts or Lunch Meat/Low Fat | 117 | Animal based processed foods |
|  | G_HAM_LUNCH_REG_DHQ | Ham, Cold Cuts or Lunch Meat/Regular | 271 | Animal based processed foods |
|  | G_HAM_NOT_LUNCH_DHQ | Ham - Not Lunch Meat | 162 | Animal based processed foods |
|  | G_HAMB_DHQ | Beef Burgers | 250 | Cookies, and savoury foods |
|  | G_HAMB_LEAN_DHQ | Beef Burgers, Lean | 288 | Cookies, and savoury foods |
|  | G_HAMB_REG_DHQ | Beef Burgers, Regular | 288 | Cookies, and savoury foods |
|  | G_HOTDOG_DHQ | Hot Dogs | 331 | Animal based processed foods |
|  | G_HOTDOG_LOWFAT_DHQ | Hot Dogs, Turkey/Low Fat | 235 | Animal based processed foods |
|  | G_HOTDOG_REG_DHQ | Hot Dogs, Regular | 331 | Animal based processed foods |
|  | G_ICECREAM_LOWFAT_DHQ | Ice Cream/Ice Milk, Low Fat | 180 | Cookies, and savoury foods |
|  | G_ICECREAM_REG_DHQ | Regular Ice Cream | 216 | Cookies, and savoury foods |
|  | G_JELLY_DHQ | Jams and Jelly, Regular | 266 | Salads, spreads, and sauces |
|  | G_JUICE_OTHER_DHQ | Other Juice | 51 | Other UPF groups |
|  | G_LASAGNA_DHQ | Lasagna, Ravioli, Shells | 163 | Cookies, and savoury foods |
|  | G_LIQUOR_DHQ | Liquor | 231 | Other UPF groups |
|  | G_M_BEEF_STEW_DHQ | Meat Component Only - Stews/Pot Pies/Mixtures | 133 | Animal based processed foods |
|  | G_M_HOTDOG_LOWFAT_DHQ | Meat Component Only - Hot Dogs,Turkey/Low Fat | 84 | Animal based processed foods |
|  | G_M_LASAGNA_DHQ | Meat Component Only - Lasagna, Ravioli, Shells | 139 | Animal based processed foods |
|  | G_M_MEATLF_DHQ | Meat Component Only - Ground Beef Meatballs/Loaves/Mixtures | 272 | Animal based processed foods |
|  | G_M_PASTA_MEATSCE_DHQ | Meat Component Only - Meat/Fish Pasta Sauce | 85 | Animal based processed foods |
|  | G_M_PIZZA_DHQ | Meat Component Only - Pizza | 278 | Cookies, and savoury foods |
|  | G_M_SAUSAGE_LOWFAT_DHQ | Meat Component Only - Sausage, Turkey/Low Fat | 267 | Other UPF groups |
|  | G_MACARONI_DHQ | Macaroni and Cheese | 222 | Quick breads, ready-to-eat/heat grains |
|  | G_MAPLE_SYRUP_DHQ | Maple Syrup on Pancakes, etc. | 234 | Other UPF groups |
|  | G_MARG_BRD_DIET_DHQ | Margarine, Diet on Bread | 533 | Salads, spreads, and sauces |
|  | G_MARG_BRD_NOFAT_DHQ | Margarine, Fat Free on Bread | 526 | Salads, spreads, and sauces |
|  | G_MARG_BRD_REG_DHQ | Margarine, Regular on Bread | 579 | Salads, spreads, and sauces |
|  | G_MARG_OTH_USE_DIET_DHQ | Margarine, Diet, Other Uses | 533 | Salads, spreads, and sauces |
|  | G_MARG_OTH_USE_NOFAT_DHQ | Margarine, Fat Free, Other Uses | 44 | Salads, spreads, and sauces |
|  | G_MARG_OTH_USE_REG_DHQ | Margarine, Regular, Other Uses | 533 | Salads, spreads, and sauces |
|  | G_MARG_PAN_DIET_DHQ | Margarine, Diet on Pancakes/Waffles | 279 | Salads, spreads, and sauces |
|  | G_MARG_PAN_NOFAT_DHQ | Margarine, Fat Free on Pancakes/Waffles | 215 | Salads, spreads, and sauces |
|  | G_MARG_PAN_REG_DHQ | Margarine, Regular on Pancakes/Waffles | 579 | Salads, spreads, and sauces |
|  | G_MARG_POT_DIET_DHQ | Margarine, Diet on Potatoes | 116 | Salads, spreads, and sauces |
|  | G_MARG_POT_NOFAT_DHQ | Margarine, Fat Free on Potatoes | 93 | Salads, spreads, and sauces |
|  | G_MARG_POT_REG_DHQ | Margarine, Regular on Potatoes | 125 | Salads, spreads, and sauces |
|  | G_MARG_VEG_DIET_DHQ | Margarine, Diet on Vegetables | 63 | Salads, spreads, and sauces |
|  | G_MARG_VEG_NOFAT_DHQ | Margarine, Fat Free on Vegetables | 44 | Salads, spreads, and sauces |
|  | G_MARG_VEG_REG_DHQ | Margarine, Regular on Vegetables | 77 | Salads, spreads, and sauces |
|  | G_MAYO_SALAD_DIET_DHQ | Mayonnaise, Diet on Salad | 250 | Salads, spreads, and sauces |
|  | G_MAYO_SALAD_NOFAT_DHQ | Mayonnaise, Fat Free on Salad | 84 | Salads, spreads, and sauces |
|  | G_MAYO_SALAD_REG_DHQ | Mayonnaise, Regular on Salad | 680 | Salads, spreads, and sauces |
|  | G_MAYO_SAND_DIET_DHQ | Mayonnaise, Diet on Sandwich | 594 | Salads, spreads, and sauces |
|  | G_MAYO_SAND_NOFAT_DHQ | Mayonnaise, Fat Free on Sandwich | 84 | Salads, spreads, and sauces |
|  | G_MAYO_SAND_REG_DHQ | Mayonnaise, Regular on Sandwich | 389 | Salads, spreads, and sauces |
|  | G_MEAL_REPL_BAR_DHQ | Meal Replacement Bars | 426 | Quick breads, ready-to-eat/heat grains |
|  | G_MEAL_REPL_LQD_DHQ | Meal Replacement Liquid | 138 | Artificial and sugar-sweetened drinks |
|  | G_MEATLF_DHQ | Ground Beef Meatballs/Loaves/Mixtures | 260 | Animal based processed foods |
|  | G_MILK_EVAP_COF_DHQ | Milk, Evaporated/Condensed in Coffee and Tea | 134 | Milk shakes, sweets, and condiments |
|  | G_MILK_SOY_CER_DHQ | Milk, Soy on Cereal | 43 | Milk shakes, sweets, and condiments |
|  | G_MILK_SOY_COF_DHQ | Milk, Soy in Coffee and Tea | 30 | Milk shakes, sweets, and condiments |
|  | G_MILK_SOY_NOT_COF_DHQ | Milk, Soy Not in Coffee, Tea, or Cereal | 43 | Milk shakes, sweets, and condiments |
|  | G_MILKSHAKE_DHQ | Milkshakes/Sodas | 97 | Milk shakes, sweets, and condiments |
|  | G_MISC_SYRUP_DHQ | Miscellaneous Syrups and Toppings | 265 | Other UPF groups |
|  | G_MUFFIN_LOWFAT_DHQ | Muffins and Dessert Bread, Low Fat | 115 | Quick breads, ready-to-eat/heat grains |
|  | G_MUFFIN_REG_DHQ | Muffins and Dessert Breads, Regular | 153 | Quick breads, ready-to-eat/heat grains |
|  | G_ORANGJCE_DHQ | Orange/Grapefruit Juice, All | 48 | Artificial and sugary-sweetened drinks |
|  | G_PANCAKE_DHQ | Pancakes, Waffles, and French Toast | 279 | Cookies, and savoury foods |
|  | G_PASTA_FA_DHQ | Pasta - Fat Added | 157 | Quick breads, ready-to-eat/heat grains |
|  | G_PASTA_MEATSCE_DHQ | Pasta with Meat/Fish Sauce | 123 | Quick breads, ready-to-eat/heat grains |
|  | G_PASTA_REDSCE_DHQ | Pasta with Meatless Red Sauce | 102 | Quick breads, ready-to-eat/heat grains |
|  | G_PIE_CUSTARD_DHQ | Pies, Cream/Custard/Other | 172 | Cookies, and savoury foods |
|  | G_PIE_FRUIT_DHQ | Pies, Fruit | 316 | Cookies, and savoury foods |
|  | G_PIE_PECAN_DHQ | Pies, Pecan | 407 | Cookies, and savoury foods |
|  | G_PIE_PUMPKIN_DHQ | Pies, Pumpkin/Sweet Potato | 243 | Cookies, and savoury foods |
|  | G_PIZZA_MEAT_DHQ | Pizza with Meat | 267 | Cookies, and savoury foods |
|  | G_PIZZA_NO_MEAT_DHQ | Pizza without Meat | 210 | Cookies, and savoury foods |
|  | G_POPCORN_DHQ | Popcorn | 538 | Cookies, and savoury foods |
|  | G_POT_FR_DHQ | Potatoes - Fried | 532 | Cookies, and savoury foods |
|  | G_POT_SALAD_DHQ | Potato Salad | 178 | Salads, spreads, and sauces |
|  | G_POULTRY_GROUND_DHQ | Ground Chicken/Turkey | 201 | Animal based processed foods |
|  | G_PRETZEL_DHQ | Pretzels, All | 384 | Cookies, and savoury foods |
|  | G_PUDDING_DHQ | Puddings and Custards | 142 | Cookies, and savoury foods |
|  | G_RICE_FA_DHQ | Rice and Grains - Fat Added | 417 | Quick breads, ready-to-eat/heat grains |
|  | G_SACCHARINE_COF_DHQ | Saccharine in Coffee and Tea | 360 | Artificial and sugary-sweetened drinks |
|  | G_SALAD_DRESS_LOWFAT_DHQ | Salad Dressing, Low Fat on Salad and Vegetables | 160 | Salads, spreads, and sauces |
|  | G_SALAD_DRESS_NOFAT_DHQ | Salad Dressing, Nearly Non-fat on Salad and Vegetables | 107 | Salads, spreads, and sauces |
|  | G_SALAD_DRESS_REG_DHQ | Salad Dressing, Regular on Salad and Vegetables | 430 | Salads, spreads, and sauces |
|  | G_SAUSAGE_DHQ | Sausage | 325 | Other UPF groups |
|  | G_SAUSAGE_LOWFAT_DHQ | Sausage, Turkey/Low Fat | 199 | Other UPF groups |
|  | G_SAUSAGE_REG_DHQ | Sausage, Regular | 325 | Other UPF groups |
|  | G_SODA_CAFF_DIET_DHQ | Soft Drinks, Diet/Caffeinated | 2 | Artificial and sugary-sweetened drinks |
|  | G_SODA_CAFF_REG_DHQ | Soft Drinks, Regular/Caffeinated | 51 | Artificial and sugary-sweetened drinks |
|  | G_SODA_DECAF_REG_DHQ | Soft Drinks, Regular/Decaffeinated | 41 | Artificial and sugary-sweetened drinks |
|  | G_SODA_DIET_DHQ | Soda, Diet | 0 | Artificial and sugary-sweetened drinks |
|  | G_SODA_REG_DHQ | Soda, Regular | 59 | Artificial and sugary-sweetened drinks |
|  | G_SOUP_CREAMED_DHQ | Soups, Creamed | 85 | Milk shakes, sweets, and condiments |
|  | G_SOUP_NOODLE_RICE_DHQ | Soups, Broth with Noodles/Rice | 67 | Quick breads, ready-to-eat/heat grains |
|  | G_TOFU_DHQ | Tofu and Soy Meats | 334 | Other UPF groups |
|  | G_WHITESCE_DHQ | White Sauce | 140 | Salads, spreads, and sauces |
|  | G_YOGURT_FRZ_DHQ | Frozen Yogurt, Ices, Sorbet | 127 | Milk shakes, sweets, and condiments |

**Supplementary Table 2**: Distribution of baseline characteristics before and after multiple imputation analysis

| Variables | Complte-case data(%) | Missing data(%) | | Imputed data(%) | |
| --- | --- | --- | --- | --- | --- |
| Cigarette smoking |  |  | |  | |
| Never | 46560 (48.2) | 13(0.00) | | 91,082 (47.8) | |
| Current | 8617 (8.9) |  |  | 18,602 (9.7) | |
| Former | 41417 (42.9) |  |  | 80,735 (42.4) | |
| BMI at baseline |  |  | |  | |
| <18.5 | 648 (0.7) | 1244(1.3) | | 1,028 (0.8) | |
| 18.5-24.99 | 32585 (34.2) |  |  | 44,558(33.5) | |
| 25-29.99 | 40538 (42.5) |  |  | 56,717(42.6) | |
| ≥30 | 21592 (22.6) |  |  | 30,670 (23.1) | |
| Educational status |  |  | | 185 | |
| Up to high school or less | 27987 (29.0) | 185(0.2) | | 73,313(29.5) | |
| Post-high school training | 33139 (34.4) |  |  | 83,402(33.5) | |
| College graduate | 35296 (36.6) |  |  | 91,280(36.8) | |
| Occupational status |  |  | |  | |
| Homemaker | 11592 (12.1) | 434(0.5) | | 28,984 (8.8) | |
| Employed | 38340 (39.9) |  | | 122,726 (37.6) | |
| Retired | 41624 (43.3) |  | | 161,398 (49.4) | |
| Others^π^ | 4617 (4.8) |  | | 12,985(3.8) | |
| Race |  |  | |  | |
| Non-Hispanic White | 88013 (91.1) | 37(0.04) | | 98,751 (90.8) | |
| Non-Hispanic Black | 3061 (3.2) |  | | 3,292 (3.0) | |
| Hispanic | 1374 (1.4) |  | | 1,875 (1.7) | |
| Asian, Pacific Islander and American Indian | 4124 (4.3) |  | | 4,817(4.4) | |
| Hispanic |  |  | |  | |
| Yes | 1,485 (1.6) | 2204(2.3) | | 2,339 (1.8) | |
| No | 92,917 (98.4) |  | | 122,966 (98.2) | |
| Marrital status |  |  | |  | |
| Married | 75771 (78.6) | 172(0.2) | | 82,543 (78.4) | |
| Widowed | 7837 (8.1) |  | | 8,534(8.1) | |
| Divorced | 9095 (9.4) |  | | 10,039(9.5) | |
| Separate/never married | 3732 (3.9) |  | | 4150(3.8) | |
| Family history of lung cancer | |  | |  | |
| No | 83499 (87.1) | 739(0.8) | | 336,421 (86.0) | |
| Yes, family member | 10058 (10.5) |  | | 42,232 (10.8) | |
| Yes, relatives | 2311 (2.4) |  | | 12,225 (3.1) | |
| Hypertension |  |  | |  | |
| No | 64,957(67.6) | 476(0.5) | | 68,320 (67.4) | |
| Yes | 31174 (32.4) |  | | 33,045 (32.6) | |
| Diabetes mellitus |  |  | |  | |
| No | 89,783(93.4) | 497(0.6) | | 106,265 (93.3) | |
| Yes | 6327 (6.6) |  | | 7,631 (6.7) | |
| Chronic bronchitis |  |  | |  | |
| No | 92,008(95.8) | 529(0.6) | | 135,486 (93.6) | |
| Yes | 4070 (4.2) |  | | 9,264 (6.4) | |
| Emphysema |  |  | |  | |
| No | 94,132(97.9) | 492(0.5) | | 132,816(96.9) | |
| Yes | 1983 (2.1) |  | | 4,249 (3.1) | |
| Polyps |  |  | |  | |
| No | 92015(93.3) | 577(0.5) | | 149,252(91.9) | |
| Yes | 6415 (6.7) |  | | 13,155(8.1) | |
| Family income (n=69,978**)** | |  | |  | |
| <$20,000 | 7,617(10.9) | 26,629(27.6) | | 28,970(11.6) | |
| $20000-49000 | 27,469(39.2) |  | | 99,277(39.7) | |
| $50,000-99,000 | 19,530(27.9) |  | | 68,687(27.4) | |
| ≥$100,000 | 6,299(9.0) |  | | 21,790(8.7) | |
| Unknown | 9,063 (12.9) |  | | 31,469(12.6) | |
| Moderate activitiy (n= 73,605) | | | 23,002(23.8) | |  |
| < 15 minutes | 21,121(28.7) |  | | 55,153(29.2) | |
| 15-29 minutes | 24,712(33.6) |  | | 63,356(33.6) | |
| ≥ 30 minutes | 27,772(37.7) |  | | 70,106(37.2) | |
| Strenuous activitiy (n=73,978) | |  | |  | |
| < 15 minutes | 33,535(45.3) | 22,629 (23.4) | | 120,466 (52.2) | |
| 15-29 minutes | 18,007(24.3) |  | | 77,013(33.4) | |
| ≥ 30 minutes | 22,526 (30.4) |  | | 33,179(14.4) | |

^π^Others includes unemployed, disabled, and extended sick leave^.^

| **Supplementary Table 3:** Cumulative incidence and competing risk regression analysis for mortality caused by CRDs overall, COPD and lung cancer among older adults participated in PLCO trial, USA. | | | | | | | |  |
| --- | --- | --- | --- | --- | --- | --- | --- | --- |
|  | **Overall CRDs mortality** | | | | | | |  |
| Characteristics | 5-year cumulative incidence (%) | p-value^1^ | 10-year cumulative incidence (%) | p-value^1^ | 20-year cumulative incidence (%) | p-value^1^ | CRR^2^  HR (95% CI) |  |
| UPF consumption |  | <0.001 |  | <0.001 |  | <0.001 |  |  |
| First quintile | 0.73 (0.61, 0.88) |  | 2.1 (2.02, 2.31) |  | 5.84 (5.66, 6.48) |  | Ref |  |
| Second quintile | 0.74 (0.62, 0.87) |  | 2.03 (1.94, 2.35) |  | 6.25 (5.92, 6.67) |  | 0.95(0.85, 1.05) |  |
| Third quintile | 0.88 (0.76, 1.02) |  | 2.4 (2.2, 2.57) |  | 6.6 (6.32, 7.13) |  | 0.98(0.88, 1.09) |  |
| Fourth quintile | 0.94 (0.83, 1.28) |  | 2.46 (2.32, 2.78) |  | 7.17 (6.76, 7.56) |  | 1.03(0.93,1.14) |  |
| Fifth quintile | 1.08 (0.92, 1.30) |  | 2.76 (2.64, 3.07) |  | 7.22 (6.74, 7.69) |  | 1.07(1.01, 1.19) ^*^  p-trend = 0.045 |  |
|  | **COPD mortality** | | | | | | |  |
| UPF consumption |  | <0.001 |  | <0.001 |  | <0.001 |  |  |
| First quintile | 0.16 (0.11, 0.23) |  | 0.48 (0.39, 0.59) |  | 1.8 (1.6, 2.1) |  | Ref |  |
| Second quintile | 0.16 (0.11, 0.22) |  | 0.52 (0.42, 0.63) |  | 2.0 (1.7, 2.3) |  | 1.02(0.86, 1.21) |  |
| Third quintile | 0.25 (0.18, 0.33) |  | 0.60 (0.49, 0.72) |  | 2.3 (2.0, 2.6) |  | 1.09(0.92, 1.29) |  |
| Fourth quintile | 0.24 (0.17, 0.31) |  | 0.73 (0.62, 0.86) |  | 2.4(2.1, 2.7) |  | 1.20(1.02, 1.41) ^*^ |  |
| Fifth quintile | 0.29(0.22, 0.38) |  | 0.78 (0.65, 0.91) |  | 2.5 (2.2, 2.8) |  | 1.20(1.02, 1.42) ^*^  p-trend = 0.014 |  |
|  | **Lung cancer mortality** | | | | | | |  |
| UPF consumption |  | 0.011 |  | 0.011 |  | 0.011 |  |  |
| First quintile | 0.41 (0.33, 0.51) |  | 1.1(0.98, 1.3) |  | 2.4 (2.1, 2.6) |  | Ref |  |
| Second quintile | 0.43 (0.34, 0.53) |  | 1.0 (0.86, 1.2) |  | 2.4 (2.1, 2.7) |  | 0.90(0.78, 1.04) |  |
| Third quintile | 0.48 (0.39, 0.59) |  | 1.2 (1.1, 1.4) |  | 2.6 (2.3, 2.9) |  | 0.93(0.81, 1.07) |  |
| Fourth quintile | 0.49 (0.40, 0.60) |  | 1.2 (1.1, 1.4) |  | 2.8(2.6, 3.1) |  | 0.99(0.86, 1.13) |  |
| Fifth quintile | 0.50 (0.41, 0.61) |  | 1.4 (1.2, 1.6) |  | 2.7 (2.5, 3.0) |  | 0.96(0.83, 1.10)  p-trend = 0.57 |  |
| *Significant at p-value < 0.05; ^1^Gray’s Test, COPD: Chronic Obstructive Pulmonary Diseases; CRDs: Chronic Respiratory Diseases; CRR= Competing Risk Regression, UPF= Ultra-Processed Food; HR: Sub-distributional Hazard Ratio.  ^2^The full model was adjusted for age, sex, arm, marital status, race, educational status, occupation, smoking, BMI, comorbidities, family history of lung cancer, and dietary energy. | | | | | | | | |

**Supplementary Table 4**: Sensitivity analysis on the association between proportion of UPF consumption and risk of mortality from overall CRDs, COPD and lung cancer among older adults participated in PLCO trial in USA.

|  | | Quintiles of proportion of UPF (%gm/day) in the total diet | | | | | | | | | | |
| --- | --- | --- | --- | --- | --- | --- | --- | --- | --- | --- | --- | --- |
|  |  | Q1(lowest) | | Q2 | | | Q3 | | Q4 | Q5(highest) | | *P* for trend |
|  |  | Ref | | HR (95% CI) | | | HR (95% CI) | | HR (95% CI) | HR (95% CI) | |  |
| **Imputed data(n=96607)** |  | | | | |  | |  | | |  |  |
| **All-cause mortality** |  | | | | |  | |  | | |  |  |
| Number of participants | | 19,622 | | 19,440 | | | 19,482 | | 19,552 | 18,511 | |  |
| Person-years | | 302654.42 | | 297144.68 | | | 293575.11 | | 292035.3 | 273889.86 | |  |
| Number of deaths | | 5774 | | 5957 | | | 6293 | | 6413 | 6186 | |  |
| Mortality rate /1000 | | 19.1 | | 20.0 | | | 21.4 | | 21.9 | 22.6 | |  |
| Unadjusted model | | Ref | | 1.06(1.02,1.09) | | | 1.14(1.10, 1.18) | | 1.17(1.13,1.21) | 1.21(1.17,1.26) | | <0.0001 |
| Age-sex adjusted model | | Ref | | 1.03(0.99,1.07) | | | 1.09(1.06, 1.14) | | 1.15(1.10,1.19) | 1.33(1.28, 1.38) | | <0.0001 |
| Multivariable adjusted model * | | Ref | | 1.0(0.96, 1.04) | | | 1.06(1.02,1.10) | | 1.07(1.03,1.11) | 1.18(1.13, 1.22) | | <0.0001 |
| Multivariable Adjusted model^µ^ | | Ref | | 1.01(0.98,1.06) | | | 1.07(1.04, 1.11) | | 1.11(1.07, 1.14) | 1.24(1.19, 1.28) | | <0.0001 |
| **Overall CRDs mortality** | |  | |  | | |  | |  |  | |  |
| Number of deaths | | 782 | | 781 | | | 849 | | 942 | 935 | |  |
| Mortality rate/1000 | | 2.6 | | 2.6 | | | 2.8 | | 3.2 | 3.4 | |  |
| Unadjusted model | | Ref | | 1.00(0.92, 1.09) | | | 1.09(1.00, 1.19) | | 1.20(1.10,1.30) | 1.28(1.17, 1.39) | | <0.0001 |
| Age-sex adjusted model | | Ref | | 0.96(0.88,1.06) | | | 1.04(0.95, 1.13) | | 1.14(1.05,1.24) | 1.34(1.22,1.46) | | <0.0001 |
| Multivariable adjusted model * | | Ref | | 0.97(0.89, 1.06) | | | 1.05(0.96, 1.14) | | 1.15(1.05,1.25) | 1.35(1.24, 1.47) | | 0.008 |
| Multivariable Adjusted model^µ^ | | Ref | | 0.96(0.87, 1.05) | | | 1.02(0.92,1.11) | | 1.09(1.00,1.20) | 1.26(1.16, 1.39) | | 0.002 |
| **Lung cancer mortality** | |  | |  | | |  | |  |  | |  |
| Number of deaths | | 391 | | 381 | | | 426 | | 462 | 467 | |  |
| Mortality rate /1000 | | 1.3 | | 1.3 | | | 1.4 | | 1.6 | 1.6 | |  |
| Unadjusted model | | Ref | | 0.97(0.84, 1.11) | | | 1.07(0.94, 1.23) | | 1.18(1.04,1.35) | 1.23(1.08, 1.41) | | < 0.001 |
| Age and sex adjusted model^¥^ | | Ref | | 0.92(0.80,1.06) | | | 1.00(0.88, 1.15) | | 1.10(0.96,1.26) | 1.21(1.05, 1.38) | | 0.007 |
| Multivariable adjusted model * | | Ref | | 0.94(0.82,1.08) | | | 1.03(0.90, 1.18) | | 1.15(1.00,1.31) | 1.25(1.09, 1.44) | | 0.001 |
| Multivariable Adjusted model^µ^ | | Ref | | 0.93(0.81,1.08) | | | 0.99(0.86,1.14) | | 1.09(0.95,1.25) | 1.16(1.01, 1.34) | | 0.042 |
| **COPD mortality** | |  | |  | | |  | |  |  | |  |
| Number of deaths | | 258 | | 278 | | | 312 | | 348 | 340 | |  |
| Mortality rate/10000 | | 9.2 | | 9.9 | | | 11.2 | | 12.5 | 13.0 | |  |
| Unadjusted model | | Ref | | 1.08(0.92, 1.27) | | | 1.22(1.04, 1.44) | | 1.39(1.19,1.62) | 1.47(1.26, 1.72) | | <0.000 |
| Age and sex adjusted model | | Ref | | 1.06(0.90,1.25) | | | 1.20(1.02,1.41) | | 1.38(1.18,1.62) | 1.63(1.39, 1.91) | | <0.000 |
| Multivariable adjusted model * | | Ref | | 1.05(0.89,1.24) | | | 1.19(1.01, 1.40) | | 1.35(1.15,1.58) | 1.67(1.42, 1.96) | | 0.004 |
| Multivariable Adjusted model^µ^ | | Ref | | 1.00(0.84,1.17) | | | 1.09(0.93,1.28) | | 1.18(1.01,1.38) | 1.45(1.22, 1.70) | | 0.005 |
| **Sensitivity analysis** | |  | |  | | |  | |  |  | |  |
| **Subjects without diabetes** | |  | |  | | |  | |  |  | |  |
| **Overall CRDs mortality (cases /cohort = 3,801 / 85,512)** | | | | | | | | |  |  | |  |
| Multivariable adjusted model | | Ref | | 0.95(0.85,1.05) | | | 1.03(0.93,1.15) | | 1.11(1.00,1.23) | 1.19(1.07, 1.320) | | <0.000 |
| **COPD mortality (cases/cohort: 1,429/85,512)** | | | | | | |  | |  |  | |  |
| Multivariable adjusted model | | Ref | | 0.98(0.82,1.17) | | | 1.08(0.91, 1.28) | | 1.04(0.87,1.23) | 1.28(1.08, 1.53) | | 0.005 |
| **Lung cancer mortality (cases/cohort: 1,866/85,512)** | | | | | | |  | |  |  | |  |
| Multivariable adjusted model | | Ref | | 0.85(0.74, 0.99) | | | 0.91(0.79, 1.06) | | 0.97(0.84,1.12) | 0.94(0.81, 1.09) | | 0.98 |
| **All-cause mortality (cases/cohort: 25,453/85,512)** | | | | | | |  | |  |  | |  |
| Multivariable adjusted model | | | Ref | | 1.01(0.97,1.05) | | 1.06(1.01, 1.09) | | 1.08(1.04,1.12) | 1.19(1.14, 1.24) | | 0.0001 |
| **Non-hypertensive subjects** | | |  | |  | |  | |  |  | |  |
| **Overall CRDs mortality (cases /cohort: 2,678/61,917)** | | | | | | |  | |  |  | |  |
| Multivariable adjusted model | | | Ref | | 0.98(0.86, 1.10) | | 1.05(0.93,1.20) | | 1.15(1.02,1.30) | 1.20(1.06,1.36) | | 0.002 |
| **COPD mortality (cases/cohort: 955/61,917)** | | | | | | |  | |  |  | |  |
| Multivariable adjusted model | | | Ref | | 0.98(0.79,1.22) | | 1.09(0.88,1.34) | | 1.12(0.91,1.38) | 1.24(1.00,1.54) | | 0.022 |
| **Lung cancer mortality (cases/cohort: 1,376/ 61,917)** | | | | | | |  | |  |  | |  |
| Multivariable adjusted model | | | Ref | | 0.89(0.75,1.06) | | 0.93(0.78,1.10) | | 0.97(0.82,1.14) | 0.95(0.79,1.12) | | 0.87 |
| **All-cause mortality (cases/cohort: 25,453/85,512)** | | | | | | |  | |  |  | |  |
| Multivariable adjusted model | | | Ref | | 1.02(0.97,1.07) | | 1.03(0.98,1.08) | | 1.09(1.03,1.14) | 1.19(1.13,1.25) | | 0.0001 |
| **Subjects without Obesity** | | |  | |  | |  | |  |  | |  |
| **Overall CRDs mortality (cases /cohort: 3,218/70,470)** | | | | | | |  | |  |  | |  |
| Multivariable adjusted model | | | Ref | | 0.99(0.88,1.11) | | 1.07(0.96, 1.21) | | 1.17(1.05, 1.31) | 1.23(1.09,1.37) | | 0.0001 |
| **COPD mortality (cases/cohort: 1,219/70,470)** | | | | | | |  | |  |  | |  |
| Multivariable adjusted model | | | Ref | | 1.01(0.83,1.23) | | 1.15(0.96, 1.39) | | 1.16(0.97,1.39) | 1.32(1.10, 1.60) | | 0.001 |
| **Lung cancer mortality (cases/cohort: 1,376/ 61,917)** | | | | | | |  | |  |  | |  |
| Multivariable adjusted model | | | Ref | | 0.91(0.77,1.06) | | 0.96(0.82,1.12) | | 0.99(0.85,1.16) | 0.98(0.84, 1.15) | | 0.75 |
| **All-cause Mortality (cases/cohort: 21,501/70,470)** | | | | | | |  | |  |  | |  |
| Multivariable adjusted model | | | Ref | | 1.02(0.97,1.06) | | 1.06(1.02,1.11) | | 1.09(1.04,1.14) | 1.20(1.15, 1.26) | | 0.0001 |
| **Subjects without Emphysema** | | |  | |  | |  | |  |  | |  |
| **Overall CRDs mortality (cases /cohort: 3,524/ 89,554)** | | | | | | |  | |  |  | |  |
| Multivariable adjusted model | | | Ref | | 0.97(0.87,1.08) | | 1.03(0.93,1.16) | | 1.13(1.02,1.26) | 1.18(1.05, 1.31) | | 0.0001 |
| **COPD Mortality (cases/cohort: 1,152/89,554)** | | | | | | |  | |  |  | |  |
| Multivariable adjusted model | | | Ref | | 1.03(0.85, 1.25) | | 1.09(0.90,1.32) | | 1.13(0.93,1.36) | 1.20(0.99, 1.46) | | 0.035 |
| **Lung Cancer Mortality (cases/cohort: 1,849/ 89,554)** | | | | | | |  | |  |  | |  |
| Multivariable adjusted model | | | Ref | | 0.87(0.75,1.01) | | 0.90(0.77,1.04) | | 0.98(0.84,1.13) | 0.97(0.83, 1.12) | | 0.80 |
| **All-cause Mortality (cases/cohort: 27,506/89,554)** | | | | | | |  | |  |  | |  |
| Multivariable adjusted model | | | Ref | | 1.02(0.98, 1.06) | | 1.06(1.02, 1.10) | | 1.09(1.05,1.13) | 1.21(1.16, 1.25) | | 0.0001 |
| **Excluding cases in the first five years of Follow-up duration** | | | | | | |  | |  |  | |  |
| **Overall CRDs Mortality (cases /cohort: 3,368/ 87,994)** | | | | | | |  | |  |  | |  |
| Multivariable adjusted model | | | Ref | | 0.97(0.86,1.09) | | 1.03(0.92,1.15) | | 1.11(0.99, 1.23) | 1.18(1.06,1.32) | | 0.0001 |
| **COPD Mortality (cases/cohort: 1,343/ 87,994)** | | | | | | |  | |  |  | |  |
| Multivariable adjusted model | | | Ref | | 0.99(0.82,1.18) | | 1.07(0.89, 1.27) | | 1.09(0.92,1.31) | 1.21(1.02, 1.45) | | 0.015 |
| **Lung Cancer Mortality (cases/cohort: 1,597/ 87,994)** | | | | | | |  | |  |  | |  |
| Multivariable adjusted model | | | Ref | | 0.87(0.74, 1.03) | | 0.92(0.78, 1.08) | | 0.96(0.83,1.13) | 0.91(0.77, 1.06) | | 0.61 |
| **All-cause Mortality (cases/cohort: 27,506/89,554)** | | | | | | |  | |  |  | |  |
| Multivariable adjusted model | | | Ref | | 1.00(0.95,1.04) | | 1.06(1.02, 1.10) | | 1.07(1.02,1.11) | 1.16(1.12, 1.21) | | 0.0001 |
| **Excluding deaths from lung cancer and COPD** | | | | | | |  | |  |  | |  |
| **Overall CRDs Mortality (cases /cohort: 525/ 88,258)** | | | | | | |  | |  |  | |  |
| Multivariable adjusted model | | | Ref | | 0.99(0.75, 1.31) | | 1.06(0.80, 1.41) | | 1.20(0.91, 1.58) | 1.49(1.14, 1.95) | | 0.0014 |
| **All-cause Mortality (cases/cohort: 25,278/88,258)** | | | | | | | | |  |  | |  |
| Multivariable adjusted model | | | Ref | | 1.01(0.97, 1.06) | | 1.08(1.03, 1.12) | | 1.09(1.05, 1.13) | 1.21(1.16,1.26) | | 0.0001 |
| **Excluding deaths from lung cancer** | | | | | | |  | |  |  | |  |
| **Overall CRDs Mortality (cases /cohort: 2,068/ 89,801)** | | | | | | |  | |  |  | |  |
| Multivariable adjusted model | | | Ref | | 1.02(0.88, 1.17) | | 1.12(0.97,1.29) | | 1.17(1.02, 1.35) | 1.39(1.20,1.60) | | 0.0001 |
| **COPD Mortality (cases/cohort: 1,541/ 89,840)** | | | | | | |  | |  |  | |  |
| Multivariable adjusted model | | | Ref | | 0.97(0.82, 1.15) | | 1.10(0.93,1.29) | | 1.09(0.93,1.28) | 1.26(1.06,1.48) | | 0.002 |
| **Adjusted for Nutrient^$^** | | | | | | |  | |  |  | |  |
| **Overall CRDs mortality** | | | | | | |  | |  |  | |  |
| Alcohol intake | | | Ref | | 0.94(0.86, 1.03) | | 0.98(0.90,1.08) | | 1.01(0.92, 1.10) | 1.13(1.03, 1.24) | | 0.003 |
| Dietary sodium intake | | | Ref | | 0.94(0.86, 1.03) | | 0.99(0.90, 1.08) | | 1.01(0.92,1.11) | 1.13(1.03,1.24) | | 0.003 |
| Trans fatty acid | | | Ref | | 0.94(0.85,1.03) | | 0.98(0.89, 1.07) | | 1.00(0.91,1.10) | 1.12(1.02, 1.23) | | 0.006 |
| Total Fat | | | Ref | | 0.94(0.86,1.03) | | 0.99(0.90,1.08) | | 1.01(0.92, 1.10) | 1.13(1.03, 1.24) | | 0.004 |
| Dietary Fiber | | | Ref | | 0.90(0.82,0.99) | | 0.92(0.83,1.01) | | 0.91(0.83,1.00) | 0.98(0.89, 1.08) | | 0.80 |
| Polyunsaturated fatty acids | | | Ref | | 0.95(0.86,1.04) | | 0.99(0.90,1.08) | | 1.01(0.92,1.10) | 1.12(1.03, 1.23) | | 0.005 |
| **COPD Mortality** | | |  | |  | |  | |  |  | |  |
| Alcohol intake | | | Ref | | 0.98(0.83, 1.17) | | 1.06(0.90,1.26) | | 1.05(0.89, 1.24) | 1.26(1.07, 1.49) | | 0.006 |
| Dietary sodium intake | | | Ref | | 0.98(0.82, 1.16) | | 1.05(0.89,1.25) | | 1.03(0.87, 1.22) | 1.21(1.02,1.44) | | 0.02 |
| Trans fatty acid | | | Ref | | 0.97(0.81,1.15) | | 1.04(0.88, 1.24) | | 1.03(0.87, 1.21) | 1.23(1.04,1.45) | | 0.014 |
| Total Fat | | | Ref | | 0.97(0.82,1.15) | | 1.05(0.89, 1.24) | | 1.03(0.88,1.22) | 1.24(1.05,1.46) | | 0.01 |
| Dietary Fiber | | | Ref | | 0.91(0.77, 1.08) | | 0.95(0.80, 1.13) | | 0.90(0.76,1.07) | 1.00(0.84,1.20) | | 0.96 |
| Polyunsaturated fatty acids | | | Ref | | 0.98(0.83,1.17) | | 1.06(0.90,1.26) | | 1.05(0.89, 1.23) | 1.24(1.05,1.47) | | 0.009 |
| **Lung Cancer Mortality** | | |  | |  | |  | |  |  | |  |
| Alcohol intake | | | Ref | | 0.89(0.77, 1.03) | | 0.94(0.81,1.08) | | 0.97(0.84, 1.11) | 0.98(0.85,1.13) | | 0.79 |
| Dietary sodium intake | | | Ref | | 0.89(0.77,1.03) | | 0.94(0.81, 1.08) | | 0.97(0.85,1.12) | 0.99(0.86,1.15) | | 0.66 |
| Trans fatty acid | | | Ref | | 0.88(0.77, 1.02) | | 0.93(0.81,1.07) | | 0.96(0.83,1.11) | 0.97(0.84, 1.12) | | 0.91 |
| Total Fat | | | Ref | | 0.89(0.77, 1.03) | | 0.93(0.81,1.08) | | 0.97(0.84, 1.11) | 0.98(0.85, 1.12) | | 0.84 |
| Dietary Fiber | | | Ref | | 0.85(.73, 0.98) | | 0.86(0.75, 1.00) | | 0.87(0.75,1.00) | 0.84(0.72, 0.98) | | 0.046 |
| Polyunsaturated fatty acids | | | Ref | | 0.89(0.77, 1.03) | | 0.94(0.81,1.08) | | 0.97(0.84, 1.11) | 0.97(0.84,1.12) | | 0.86 |
|  | | |  | |  | |  | |  |  | |  |

*The full model was adjusted for age, sex, marital status, education, occupation, study arm, race, BMI, smoking, hypertension, diabetes, stroke, heart attack, chronic bronchitis, emphysema, and family history of lung cancer and total energy (kcal/day).

^µ^The model was additionally adjusted for family income and physical activity.

Alcohol intake was also adjusted for UPF consumption by omitting total energy from the model and for sensitivity analysis subjects with baseline health problem were excluded so that the model was not adjusted for that disease. ^$^Nutrients were adjusted for energy before including in the model through residual method. COPD: Chronic Obstructive Pulmonary Diseases; CRDs: Chronic Respiratory Diseases; HR: Hazard Ratio.

Supplementary Table 5: Multivariable Cox regression models for the association between UPF consumption (gm/day) and risk of mortality from CRDs overall, lung cancer and COPD adjusted for each subgroup of UPF intake, fish, and fruits and vegetables intake among adults in the USA.

| Subgroups of foods^a^ | CRDs mortality^b^ | COPD mortality^b^ | Lung cancer mortality^b^ |
| --- | --- | --- | --- |
|  | HR (95% CI) | HR (95% CI) | HR (95% CI) |
| Overall UPF intake | 1.14(1.04, 1.24) | 1.18(1.01, 1.38) | 1.02(0.89, 1.17) |
| Adjusted for UPF subgroups |  |  |  |
| Animal based processed foods | 1.12(1.02, 1.22) | 1.15(0.98, 1.34) | 0.99(0.86, 1.14) |
| Artificial and sugar-sweetened drinks | 1.08(0.98,1.18) | 1.09(0.92, 1.28) | 0.97(0.84,1.12) |
| Salads, spreads, and sauces | 1.16(1.06, 1.27) | 1.21(1.04, 1.42) | 1.04(0.90, 1.19) |
| Milk shakes, sweets, and condiments | 1.13(1.03, 1.23) | 1.18(1.01, 1.38) | 1.01(0.88, 1.16) |
| Quick breads, ready-to-eat/heat grains | 1.14(1.04,1.24) | 1.18(1.01, 1.37) | 1.02(0.89, 1.17) |
| Cookies, and savoury foods | 1.18(1.08, 1.30) | 1.27(1.08, 1.49) | 1.05(0.92, 1.22) |
| Other UPF groups | 1.15(1.04, 1.27) | 1.24(1.04, 1.48) | 1.05(0.90, 1.22) |
| Adjusted for unprocessed foods |  |  |  |
| Total fruits and vegetable | 1.12(1.02,1.22) | 1.14(1.01,1.34) | 1.00(0.87,1.14) |
| Total Fish intake | 1.14(1.04, 1.25) | 1.18(1.01, 1.37) | 1.02(0.89,1.18) |

^a^ The subgroups of UPF intake (g/day) at baseline was adjusted for energy using residual method.

^b^ The models were adjusted for age (continuous), sex(men, women), ethnicity(Hispanic, non-Hispanic), marital status (married, widowed, divorced, separated/never married), study arm (intervention, control), educational status (≤ grade12, post-high school training, college graduate) occupation (homemaker, employed, retired , others), cigarette smoking (never, current, former), BMI(<18.5, 18.5-24.99, 25-29.99, ≥30 kg/m^2^), history of lung cancer (yes, no), hypertension (yes, no), diabetes(yes, no), stroke(yes, no), emphysema (yes, no), bronchitis (yes, no) and alcohol (gm/day). Total energy intake (kcal/day) was not adjusted in these models as each subgroup of UPF were initially adjusted energy.

COPD: Chronic Obstructive Pulmonary Diseases; CRDs: Chronic Respiratory Diseases; HR: Hazard Ratio; UPF: Ultra-Processed Food

**Supplementary Table 6:** Multivariable Cox regression model for the association between UPF consumption in the total diet(%g/day) and overall CRDs mortality, COPD and Lung cancer based on the intensity and duration of cigarette smoking among older adults enrolled in the PLCO trial in USA.

|  | CRDs mortality^b^ | | COPD mortality^b^ | | Lung cancer mortality^b^ | |
| --- | --- | --- | --- | --- | --- | --- |
|  | HR (95% CI) | p-for trend | HR (95% CI) | p-for trend | HR (95% CI) | p-for trend |
| Quintile of UPF^a^ |  | < 0.0001 |  | < 0.0001 |  | 0.0003 |
| Q2 | 0.99(0.90, 1.08) |  | 1.03(0.87,1.22) |  | 0.96(0.83, 1.11) |  |
| Q3 | 1.08(0.98, 1.18) |  | 1.19(1.01, 1.40) |  | 1.05(0.91,1.21) |  |
| Q4 | 1.16(1.06, 1.27) |  | 1.31(1.11, 1.54) |  | 1.15(1.00,1.32) |  |
| Q5 | 1.34(1.22, 1.47) |  | 1.57(1.33,1.85) |  | 1.22(1.06,1.40) |  |

^a^The lowest quintile (Q1) was the reference group.

^b^Models were adjusted for age, sex, marital status, education, occupation, study arm, ethnicity, BMI, smoking, family income, physical activity, hypertension, diabetes, stroke, heart attack, chronic bronchitis, emphysema, and family history of lung cancer and total energy (kcal/day).

COPD: Chronic Obstructive Pulmonary Diseases; CRDs: Chronic Respiratory Diseases; HR: Hazard Ratio; UPF: Ultra-Processed Food.

**Supplementary Table 7:** The association between proportion of UPF consumption in the total energy(%kcal) and overall CRDs mortality, COPD and Lung cancer among older adults enrolled in the PLCO trial in USA.

|  | | Quintiles of proportion of UPF in the total diet | | | | | | | | |
| --- | --- | --- | --- | --- | --- | --- | --- | --- | --- | --- |
|  |  | Q1(lowest) | Q2 | | Q3 | | Q4 | Q5(highest) | | *P* for trend |
|  |  | Ref | HR (95% CI) | | HR (95% CI) | | HR (95% CI) | HR (95% CI) | |  |
| **All-cause Mortality** |  | | |  | |  | | |  |  |
| Number of participants | | 18,108 | 18,137 | | 18,153 | | 18,708 | 18,347 | |  |
| Person-years | | 280053.6 | 280467.8 | | 279517.0 | | 278506.5 | 261110.6 | |  |
| Number of deaths | | 5,538 | 5,488 | | 5,449 | | 5,891 | 6,334 | |  |
| Mortality rate /1000 | | 18.8 | 19.8 | | 21.3 | | 21.7 | 22.4 | |  |
| Unadjusted model | | Ref | 1.03(1.00, 1.07) | | 1.05(1.01, 1.09) | | 1.13(1.09,1.17) | 1.25(1.21, 1.30) | | <0.0001 |
| Age-sex adjusted model^¥^ | | Ref | 1.03(0.99, 1.07) | | 1.10(1.06, 1.14) | | 1.15(1.11,1.19) | 1.33(1.28, 1.38) | | <0.0001 |
| Multivariable adjusted model * | | Ref | 1.02(0.98, 1.06) | | 1.09(1.05, 1.13) | | 1.13(1.09,1.17) | 1.28(1.23,1.33) | | <0.0001 |
| **Overall CRDs mortality** | |  |  | |  | |  |  | |  |
| Number of deaths | | 631 | 686 | | 763 | | 880 | 1,132 | |  |
| Mortality rate/1000 | | 2.3 | 2.5 | | 2.8 | | 3.1 | 4.1 | |  |
| Unadjusted model | | Ref | 1.08(0.97, 1.21) | | 1.22(1.09, 1.35) | | 1.37(1.24,1.52) | 1.86(1.69, 2.05) | | <0.0001 |
| Age-sex adjusted model^¥^ | | Ref | 1.09(0.98, 1.21) | | 1.22(1.10, 1.35) | | 1.38(1.24,1.53) | 1.83(1.66, 2.01) | | <0.0001 |
| Multivariable adjusted model * | | Ref | 1.05(0.95,1.17) | | 1.08(0.97, 1.19) | | 1.15(1.03,1.27) | 1.30(1.17, 1.43) | | <0.0001 |
| **Lung Cancer Mortality** | |  |  | |  | |  |  | |  |
| Number of deaths | | 321 | 345 | | 385 | | 425 | 539 | |  |
| Mortality rate /1000 | | 1.3 | 1.3 | | 1.4 | | 1.6 | 1.6 | |  |
| Unadjusted model | | Ref | 1.07(0.92, 1.25) | | 1.21(1.04, 1.40) | | 1.30(1.13,1.51) | 1.73(1.51,1.99) | | <0.0001 |
| Age and sex adjusted model^¥^ | | Ref | 1.07(0.91, 1.24) | | 1.19(1.03, 1.38) | | 1.29(1.11,1.49) | 1.69(1.47, 1.94) | | <0.0001 |
| Multivariable adjusted model * | | Ref | 1.03(0.88, 1.19) | | 1.05(0.90, 1.21) | | 1.06(0.91,1.23) | 1.19(1.03, 1.37) | | 0.023 |
| **COPD Mortality** | |  |  | |  | |  |  | |  |
| Number of deaths | | 210 | 245 | | 281 | | 338 | 462 | |  |
| Mortality rate/10000 | | 9.2 | 9.9 | | 11.2 | | 12.5 | 13.0 | |  |
| Unadjusted model | | Ref | 1.16(0.97, 1.40) | | 1.35(1.13, 1.61) | | 1.59(1.34,1.89) | 2.30(1.95, 2.70) | | <0.0001 |
| Age and sex adjusted model | | Ref | 1.19(0.99, 1.43) | | 1.38(1.15, 1.65) | | 1.64(1.38,1.95) | 2.30(1.95,2.71) | | <0.0001 |
| Multivariable adjusted model * | | Ref | 1.12(0.93, 1.35) | | 1.18(0.99, 1.42) | | 1.30(1.09,1.55) | 1.53(1.29,1.81) | | <0.0001 |

¥ Model adjusted for age (timescale variable) and sex; * Model adjusted for age, sex, marital status, education, ethnic background, study arm, occupation, history of lung cancer, hypertension, diabetes, stroke, heart attack, liver comorbidity, colon comorbidity, aspirin and ibuprofen use, cigarette smoking, alcohol intake(gm/day), BMI, emphysema, chronic bronchitis, and total dietary energy. COPD: Chronic Obstructive Pulmonary disease; CRDs: Chronic Respiratory Diseases; HR: Hazard Ratio.

1. Created in Lucid chart ([www.lucidchart.com](http://www.lucidchart.com)) available at [https://lucid.app/lucidchart/a5a16cd9-0d59-4c51-beaa-5af92e76a536/edit?page=0_0&invitationId=inv_00139298-34f5-4c94-b64a-ee314901948b#](https://lucid.app/lucidchart/a5a16cd9-0d59-4c51-beaa-5af92e76a536/edit?page=0_0&invitationId=inv_00139298-34f5-4c94-b64a-ee314901948b). [↑](#footnote-ref-1)
